# Sex-dimorphic neuroprotective effect of CD163 in an α-synuclein mouse model of Parkinson’s disease

**DOI:** 10.1038/s41531-023-00606-w

**Published:** 2023-12-13

**Authors:** Sara A. Ferreira, Conghui Li, Ida H. Klæstrup, Zagorka Vitic, Rikke K. Rasmussen, Asger Kirkegaard, Gitte U. Toft, Cristine Betzer, Pia Svendsen, Poul H. Jensen, Yonglun Luo, Anders Etzerodt, Søren K. Moestrup, Marina Romero-Ramos

**Affiliations:** 1 Department of Biomedicine, Aarhus University, Aarhus, Denmark; 2 Danish Research Institute of Translational Neuroscience – DANDRITE, Aarhus University, Aarhus, Denmark; 3 Department of Biology, University of Copenhagen, Copenhagen, Denmark; 4 Department of Molecular Medicine, University of Southern Denmark, Odense, Denmark; 5 Steno Diabetes Center Aarhus, Aarhus University Hospital, Aarhus, Denmark

**Keywords:** Parkinson's disease, Movement disorders

## Abstract

Alpha-synuclein (α-syn) aggregation and immune activation represent hallmark pathological events in Parkinson’s disease (PD). The PD-associated immune response encompasses both brain and peripheral immune cells, although little is known about the immune proteins relevant for such a response. We propose that the upregulation of CD163 observed in blood monocytes and in the responsive microglia in PD patients is a protective mechanism in the disease. To investigate this, we used the PD model based on intrastriatal injections of murine α-syn pre-formed fibrils in CD163 knockout (KO) mice and wild-type littermates. CD163KO females revealed an impaired and differential early immune response to α-syn pathology as revealed by immunohistochemical and transcriptomic analysis. After 6 months, CD163KO females showed an exacerbated immune response and α-syn pathology, which ultimately led to dopaminergic neurodegeneration of greater magnitude. These findings support a sex-dimorphic neuroprotective role for CD163 during α-syn-induced neurodegeneration.

## Introduction

Parkinson’s disease (PD) is a neurodegenerative disease classically characterized by the loss of dopaminergic neurons in the substantia nigra (SN) and the intraneuronal alpha-synuclein (α-syn) aggregation in Lewy bodies^1^. An immune response has been suggested to parallel, or even precede neurodegeneration in PD, influencing neuronal health^2^. Microgliosis and a marked pro-inflammatory profile are found in *postmortem* PD brains^3,4^ and in α-syn-based models^5–7^. This immune response involves not only brain-resident glia (microglia and astrocytes), but also entails peripheral innate and adaptive immune cells^2^. Interestingly, growing evidence support sex differences in the immune changes associated with PD that might have a significant relevance in the PD risk, presentation and progression^8–11^. However, little is known about what proteins might be involved in the differential immune response and/or which ones might exert a neuroprotective effect.

The scavenger receptor CD163 is considered a marker for anti-inflammatory macrophages. CD163 expression increases upon anti-inflammatory cytokine IL-10 or glucocorticoid exposure^12^, but also under certain inflammatory conditions, putatively as a protective mechanism^13^. Conversely, activation of pro-inflammatory receptors results in CD163 cleavage from the monocytes-membrane (producing soluble (s)CD163), thus associating membrane-bound CD163 loss with pro-inflammatory phenotypes^14^. CD163 uptakes the hemoglobin/haptoglobin complexes for lysosomal degradation^13^. CD163’s specific role in the immune system is still undefined, although studies of CD163 knockout (CD163KO) mice support an anti-inflammatory function^15,16^. Increased CD163+ cell numbers have been found in PD and Alzheimer’s disease (AD) *postmortem* brains^17,18^ and in the brain of PD models^19,20^. CD163 expression is lost during microglia development, hence absent in adult surveilling microglia^21–23^. Therefore, CD163 expression in the brain seems associated with infiltration or otherwise ectopic CD163 upregulation in microglia. Interestingly, recent single-cell (sc)RNA sequencing studies show that CD163 upregulation is associated with microglia response during AD and PD in humans^24,25^.

Our prior data supports a role for CD163-expressing cells in patients with PD and a sex-dimorphic behavior of the CD163 receptor^26^. Moreover, we have reported that a higher percentage of CD163 cells in the blood was associated with lower inflammation in the brain and better putaminal dopaminergic neurotransmission in REM sleep behavior disorder (RBD) patients, suggesting a protective function for the CD163 cells in prodromal stages of PD^27^. Lastly, we recently showed an increased number of CD163 cells and expression levels in the blood of PD patients^11^. We speculate that the observed increase in CD163 expression is part of a neuroprotective compensatory mechanism exerted by myeloid cells.

To investigate the significance of the CD163 receptor in the PD-like neurodegeneration, we analyzed α-syn neurotoxicity, pathology, and immune alterations in CD163KO mice by injecting recombinant α-syn pre-formed fibrils (PFF) into the striatum of these animals vs. wild-type (WT). In addition, we performed SMART-seq2 on microglia and macrophage populations in the brain to assess transcriptomic alterations upon CD163 deletion. We hypothesize that CD163 deficiency leads to impaired immune responses to α-syn PFF in a possible sex-dependent manner.

## Results

### Intrastriatal α-syn PFF induced long-lasting motor impairment in CD163KO males

**Fig. 1:**
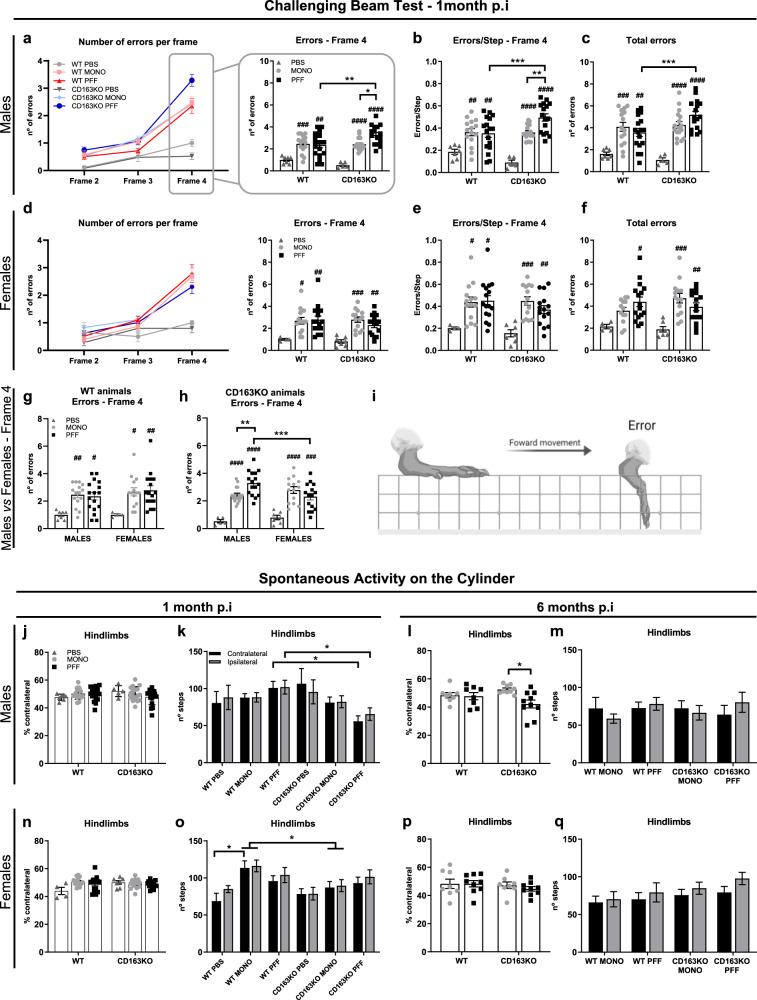
Assessment of motor performance on the Challenging Beam and the Cylinder tests. Top: line with average and bar graphs with individual points show number of errors per frame, number of errors and errors/step in Frame 4, and total number of errors (sum of frames 2–4,) in the Challenging Beam test, 1 month post-PBS and α-syn MONO/PFF unilateral injection in the Striatum of **a**–**c** males and **d**–**f** females. **g**, **h** Show error differences between males and females in WT and CD163KO animals. **i** Illustration representing the portrayal of an error during a forward movement (image created with BioRender.com). Bottom: bar graphs with/without individual points represent the contralateral hindlimb use (as percentage of total hindlimbs) and the number of hindlimb steps in the cylinder in **j**–**m** males and **n**–**q** females. Values are mean ± SEM (*n* = 4–7 (PBS group), 14–17 (MONO/PFF group) for behavior at 1 month; *n* = 8–10 for behavior at 6 months). Statistics: two-way ANOVA followed by Sidak’s multiple comparison test. *P* values are given according to the number of symbols, e.g., *p < 0.05, **<0.01, ***<0.001, ****<0.0001. # different from the corresponding PBS control.

To induce α-syn pathology, murine α-syn PFF, α-syn monomeric (MONO), or control phosphate-buffered saline (PBS) were unilaterally injected into the striatum (see Supplementary Fig. 1 for study design). Based on our prior observations regarding CD163 in humans with PD, we analyzed sexes separately^26^. To evaluate whether the genetic deletion of CD163 would lead to differential sensorimotor behavioral changes after α-syn PFF injection, mice were assessed with the challenging beam at 1 and 6 months’ post-injection (p.i.) (Fig. 1a–i and Supplementary Fig. 2). At 1 month, we observed a significant increase in the number of steps, errors, errors/step on frame 4 and total errors (the narrowest and most challenging frame), but no changes in time, in all α-syn-injected animals (males and females) when compared to their respective PBS- controls (Fig. 1a–f and Supplementary Fig. 2a, b). This suggests that not only fibrillar α-syn but also an excess of monomeric protein is sufficient to induce neuronal changes leading to motor deficits in mice. PFF-CD163KO males had significantly more errors and errors/step in frame 4 (vs. WT, PBS- and MONO-CD163KO males) (Fig. 1a, b) and more total errors (frames 2–4) (vs. PFF-WT and PBS-CD163KO males) (Fig. 1c). No changes in errors were found between different genotypes in the female groups (Fig. 1d–f). When comparing sexes, PFF-CD163KO males showed significantly more errors in frame 4 than PFF-CD163KO females (Table 1 and Fig. 1h), indicating a sex-specific role for CD163 in the early phenotypic response to α-syn induced pathology. When analyzing the temporal evolution in the motor performance, most of the readouts at 6 months remained similar to those at 1 month. PFF-CD163KO males still showed a greater number of total errors and total errors/ step than PFF-WT males after 6 months, though no differences between PFF and MONO-males were seen (Supplementary Fig. 2e). At 6 months, all PFF-injected females took longer to transverse frame 4 than at 1 month (two-way ANOVA genotype and time, *p* < 0.05), but no differences were seen among the female groups (Supplementary Fig. 2b, d, f).

**Table 1:** Sex differences in the α-syn PFF-injected groups within genotypes.

	1 month p.i		6 months p.i	
	WT	CD163KO	WT	CD163KO
	M vs. F	M vs. F	M vs. F	M vs. F
CBT-errors frame 4	=	↑M	=	=
CT-Hindlimbs	=	↓M (both paws)	=	↓M (contra paw)
MJF14	=	=	=	↑F
pSer129	–	–	=	↑F
p62	=	↓F	=	↑F
CD68	=	↓F	=	=
IBA-1	–	–	=	=
GFAP	↑F amyg	↓F str	=	=
MHCII	↑M	↑M	=	=
CD4 (ipsi. str.)	=	↓F	=	↑F
CD8 (ipsi. str.)	=	↑M	=	↑F (*p* = 0.07)
TH	=	=	=	↓F

↑ or ↓ represents increased or decreased in one sex vs the other.

“=” no difference between sexes, “–” not performed.

*WT* wild type, *CD163KO* CD163 knockout, *p.i.* post-injection, *F* female, *M* male, *CBT* Challenging Beam Test, *CT* Cylinder Test, *ipsi* ipsilateral, *str* striatum, *amyg* amygdala, *TH* tyrosine hydroxylase.

Evaluation of the spontaneous activity in the cylinder (Fig. 1j–q) at 1 month showed a general decrease in hindlimbs use in PFF-CD163KO males (vs. PFF-WT males) (Fig. 1k). MONO-WT females had more hindlimbs steps (vs. PBS-WT and MONO-CD163KO females) suggesting certain motoric consequences after α-syn-MONO injection influenced by the CD163 absence in females (Fig. 1o). When comparing sexes, MONO-WT and PFF-CD163KO females had more hindlimbs steps (contralateral and ipsilateral) than MONO-WT and PFF-CD163KO males, respectively (Table 1 and Supplementary Fig. 3a–d). At 6 months, the total paw use was similar across groups; however, PFF-CD163KO males showed paw asymmetry by using the contralateral hindlimb significantly less than MONO-CD163KO (Fig. 1l). In conclusion, both MONO and PFF α-syn injections lead to motor alterations that appeared distinct and sex-dependent in CD163-deficient mice, but not in WT mice. Collectively, these observations suggest a sex dimorphism in the CD163 system response to α-syn neuronal pathology with CD163 deletion resulting in an enhanced motor phenotype in males that had more early motor defects and paw asymmetry in long term.

### CD163KO females display enhanced α-syn pathology

**Fig. 2:**
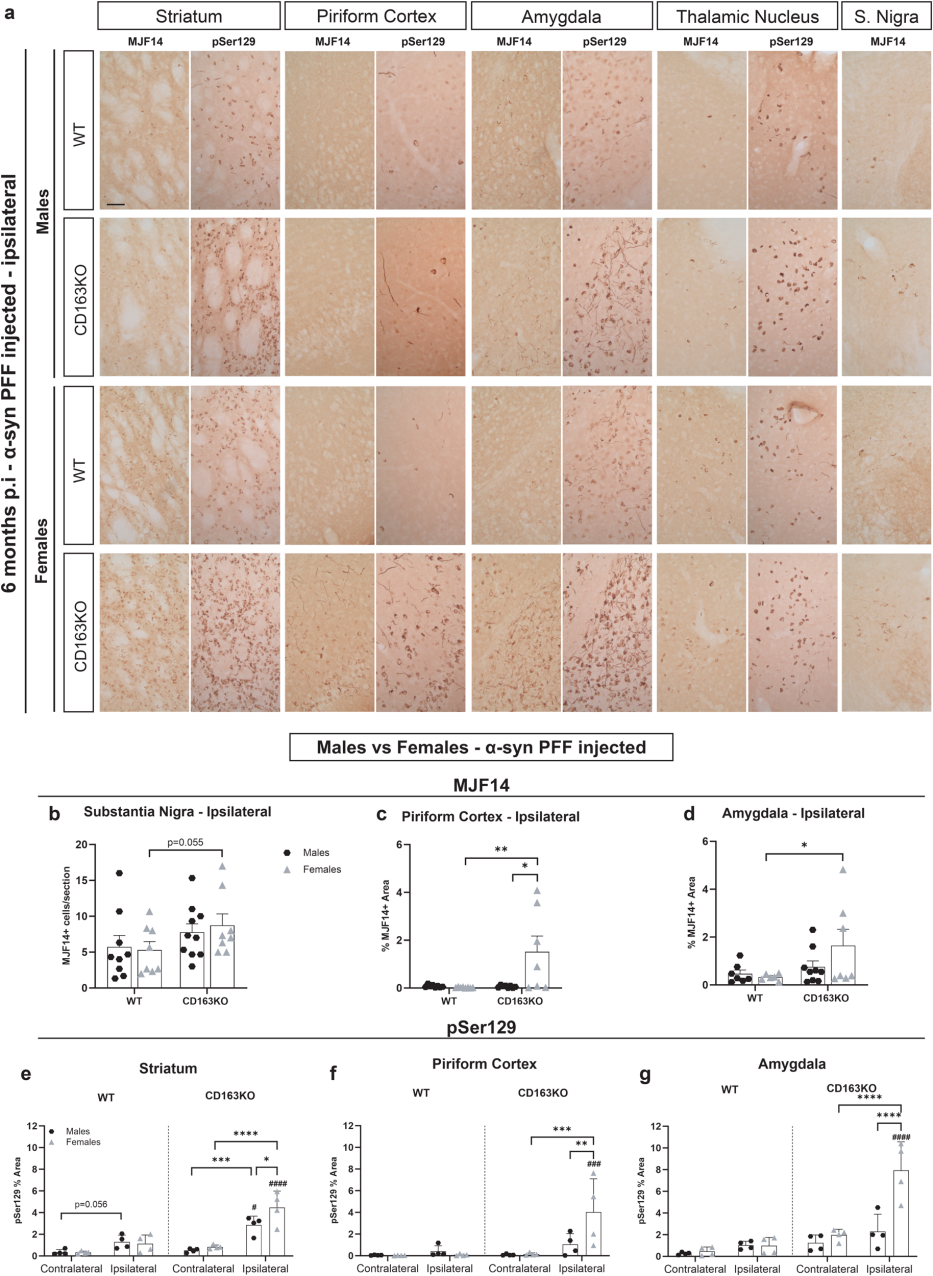
Aggregated and phosphorylated α-syn expression in the striatum and interconnected regions after α-syn PFF injection. **a** Representative images of MJF14 (left columns) and pSer129 α-syn immunostaining (right columns) in α-syn PFF animals 6 months p.i. **b** Bar graphs with individual points show the total number of cells per section in the SN, and the percentage of area covered by MJF14+ staining in the **c** ipsilateral piriform cortex and **d** ipsilateral amygdala, or pSer129+ staining in the **e** striatum, **f** piriform cortex and **g** amygdala, 6 months post-α-syn-PFF injection. Scale: 50 μm applies to all. Values are mean ± SEM (*n* = 4–10). Statistics: two-way ANOVA followed by Sidak’s multiple comparison test. *P* values are given according to the number of symbols, e.g., *p < 0.05, **<0.01, ***<0.001, ****<0.0001. # different to the corresponding WT group.

α-Syn pathology was analyzed using the MJF14 antibody, which preferentially binds aggregated α-syn^28–30^; and an anti-phosphorylated α-syn at Ser129 (pSer129) antibody, which is enriched in PD brains^31^ (Fig. 2a and Supplementary Fig. 4). At 1 month p.i, MJF14+ α-syn pathology was present in all PFF-groups in the ipsilateral hemisphere of all regions evaluated (striatum, piriform cortex, amygdala, thalamus and SN) and was mostly prevalent in neuropil-like structures (Supplementary Fig. 4a–d). No major MJF14+ pathology was seen in the contralateral hemisphere, except few structures in the cortex and amygdala (not shown). After 6 months, α-syn aggregation was increased in all regions and was seen in numerous cell-body-like structures (Fig. 2a). PFF-CD163KO females had significantly greater MJF14+ staining (vs. PFF-WT female Fig. 2b–d). pSer129 α-syn immunostaining revealed bilateral pathology in the striatum of all PFF-injected animals (vs. MONO) after 6 months (Supplementary Fig. 4e, f). pSer129 α-syn staining was substantially higher in PFF-CD163KO mice but particularly enriched in PFF-CD163KO females compared to all other groups and across all structures analyzed (Table 1, Fig. 2e–g and Supplementary Fig. 4e–j). Moreover, PFF-CD163KO females showed more pSer129 α-syn pathology in the contralateral hemisphere than PFF-WT females in all regions, and this was also true for PFF-CD163KO males in the amygdala (Supplementary Fig. 4e, g, i). No positive staining for any of the antibodies was found in PBS-injected mice and none or rare structures were found in α-syn MONO-injected animals (Supplementary Fig. 4k–n). Therefore, the absence of CD163 decreases the brain’s ability to avoid α-syn pathology resulting in more α-syn aggregation and Ser129 phosphorylation in both sexes but especially in females.

### α-syn aggregation is associated with p62 accumulation

**Fig. 3:**
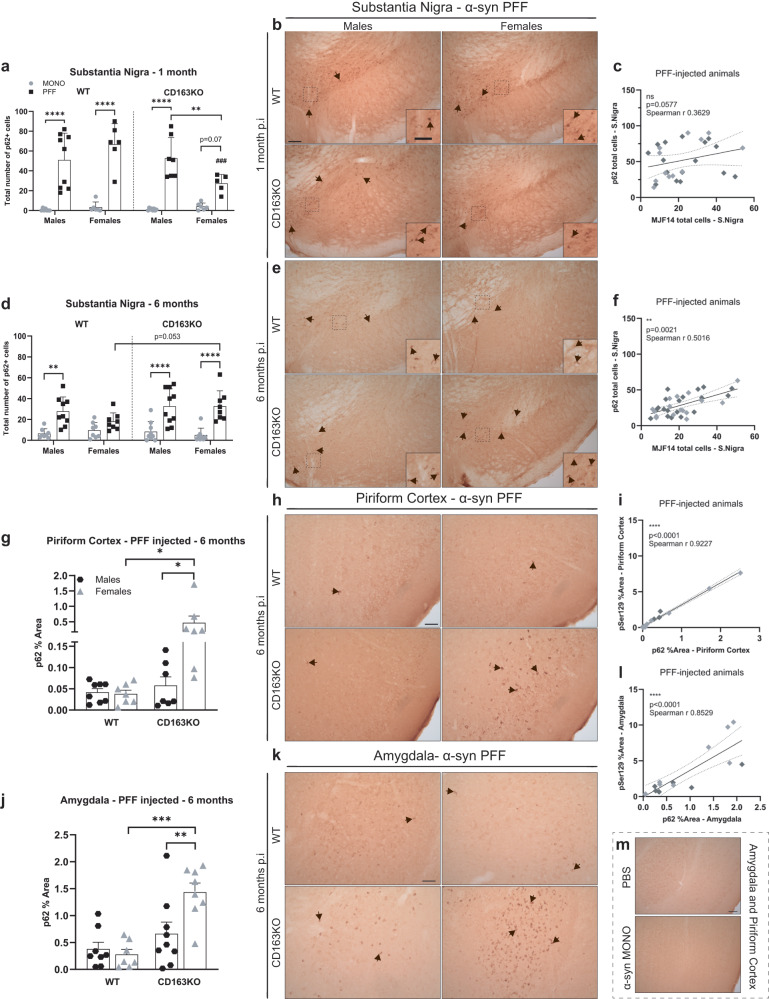
Differential expression of p62/SQSTM1 autophagy marker in the substantia nigra, piriform cortex and amygdala after α-syn PFF injection. Bar graphs with individual points illustrate the total number of p62+ cells in the SN at **a** 1 and **d** 6 months p.i. **b** Representative images of p62/SQSTM1 immunostaining in α-syn PFF animals at **b** 1- and **e** 6 months p.i. in the SN, **h** piriform cortex and **k** amygdala. **g** Bar graphs with individual points illustrate the percentage of area covered by p62+ staining 6 months after α-syn PFF-injection in WT/CD163KO males and females in the piriform cortex and **j** amygdala. The number of p62+ cells correlated to MJF14+ cells in the SN at **c** 1- and **f** 6 months p.i. The percentage of area covered by p62+ staining correlated to the one covered by pSer129+ staining **i** in the piriform cortex and **l** amygdala at 6 months p.i. in all α-syn PFF animals. In the correlation plots: light gray symbols represent females and dark gray males. **m** Representative images of p62/SQSTM1 immunostaining of amygdala and piriform cortex from PBS or α-syn MONO injected animals. Scale bar represents 100 and 50 μm in cropped images in (**b**, **e**) and 50 μm in (**h**, **k**). Values are mean ± SEM (*n* = 6–10). Statistics: Spearman two-tail *p* values (*<0.05, **<0.01, ***<0.001, ****<0.0001), Spearman *r* and best-fit slope with 95% confidence intervals are plotted. Two-way ANOVA followed by Sidak’s multiple comparison test. *p < 0.05, **<0.01, ***<0.001, ****<0.0001.

As α-syn aggregation has been associated with the accumulation of the autophagy-related protein p62/SQSTM1^32^, we performed immunohistochemistry against p62 (Fig. 3). We observed increased p62+ structures in the ipsilateral SN 1 month p.i. in all PFF-injected animals (vs. MONO). This was milder in PFF-CD163KO females, which had significantly fewer p62+ structures than CD163KO males and WT females (Table 1 and Fig. 3a, b). However, at 6 months, while p62+ structures in the SN decreased in most of the PFF-injected animals, this was not seen in the PFF-CD163KO females that tend to have higher p62 signal than WT females (*p* = 0.053) (Fig. 3d, e). Corroborating this delayed p62 accumulation PFF-CD163KO females showed significantly more p62 staining in the piriform cortex and amygdala (vs. CD163KO males and WT females) (Table 1 and Fig. 3g, h, j, k). Altogether, our data suggest that lacking CD163 results in a temporal delay of α-syn-induced p62 accumulation in females, but not in males.

p62 accumulation was associated with α-syn pathology, since the number of p62-expressing cells moderately correlated with the MJF14+ cell numbers in the SN (at 1 and 6 months) and strongly with pSer129 pathology in the piriform cortex and amygdala at 6 months in PFF-injected animals (Fig. 3c, f, i, l). Indeed, confocal analysis of p62 and MJF14 in the different brain areas at 6 months revealed that some, but not all, p62 structures colocalized with aggregated α-syn (particularly cytoplasmic perinuclear aggregates of half-moon shape) (Supplementary Fig. 5). No p62 co-localization was seen with Iba1+ microglia (Supplementary Fig. 6i) or GFAP+ astrocytes (Supplementary Fig. 7h); and no p62+ aggregates were found in PBS and MONO-injected animals (Fig. 3m). However, confocal imaging confirmed the presence of p62+ structures in tyrosine hydroxylase (TH) + dopaminergic neurons in SN (Supplementary Fig. 8a, arrowheads). Notably, p62+ structures in the SN inversely correlated to the use of the contralateral hindlimb of CD163KO males in the cylinder at 6 months (Spearman *r* = –0.734, *p* = 0.0003), suggesting that dopaminergic autophagic changes partially contribute to the motor defects seen in CD163KO males.

### Microglial response to α-syn is influenced by the lack of CD163

**Fig. 4:**
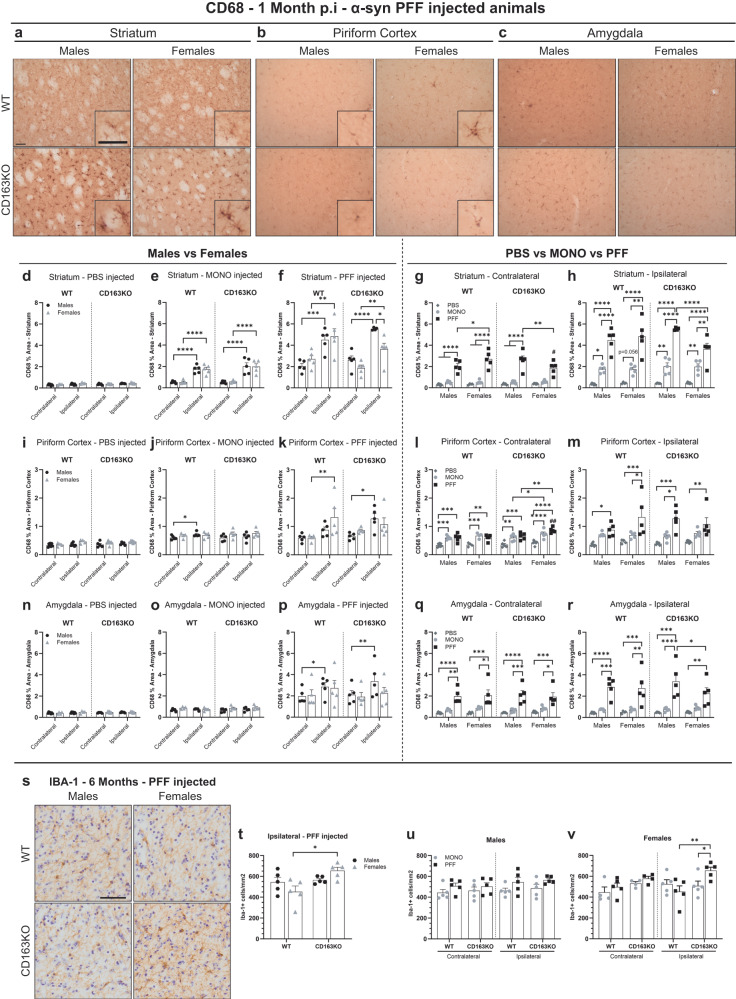
CD163KO females display dysfunctional microglial responses to α-syn PFF injection. **a** Representative images of CD68 immunostaining in α-syn PFF animals in the striatum, **b** piriform cortex and **c** amygdala at 1 month p.i. Scale: 50 μm applies to all. Bar graphs with individual points illustrate the percentage of area covered by CD68+ staining after PBS, α-syn MONO and PFF injection in the **d**–**h** striatum, **i**–**m** piriform cortex and **n**–**r** amygdala at 1 month p.i. **s** Representative ipsilateral images of Iba-1 immunostaining in α-syn PFF-injected animals 6 months p.i. Scale: 50 μm applies to all in (**s**). **t** Bar graphs with individual points show the number of Iba-1 positive cells per mm^2^ in the ipsilateral SN, 6 months after α-syn PFF injection. **u**–**v** and in the contralateral and ipsilateral SN, 6 months after α-syn MONO and PFF injection. Values are mean ± SEM (*n* = 5). Statistics: two-way ANOVA followed by Sidak’s multiple comparison test, or **m** with Bonferroni corrections applied (i.e. *p < 0.0125). *P* values are given according to the number of symbols, e.g., *p < 0.05, **<0.01, ***<0.001, ****<0.0001. # different to the corresponding WT group.

To evaluate changes in the immune response due to CD163 deletion, we performed immunohistochemistry for different immune-relevant proteins. CD68 expression was used as an indirect measurement of immune phagocytic activity and analyzed in the striatum, piriform cortex, amygdala, and SN (Fig. 4a–r and Supplementary Fig. 6a–h). At 1 month, CD68 expression in PBS-injected animals was very low and similar across hemispheres and groups in all areas except in the ipsilateral SN, which was increased (Fig. 4d, i, n and Supplementary Fig. 6a). Thus, while the surgery itself was not sufficient to induce overt myeloid phagocytic activity in the brain, the immune cells in the SN had a higher sensibility to mild-innocuous changes in anatomically connected areas. In general, although not always significant, CD68 expression in MONO-injected brains was bilaterally higher than in PBS animals (Fig. 4d–r and Supplementary Fig. 6a–e). All MONO mice, irrespective of sex and genotype, showed significant ipsilateral CD68 upregulation in the striatum (Fig. 4e, h). Therefore, an excess of soluble α-syn is sufficient to induce phagocytic activity in myeloid cells in the brain.

PFF-animals showed robust bilateral CD68 upregulation in all areas examined (vs. PBS) (Fig. 4a–r and Supplementary Fig. 6c–e) that was higher than the one seen in MONO groups (Fig. 4g, h, m, q, r). CD68 upregulation was greater in the ipsilateral side (vs. contralateral) in all PFF-animals in the striatum (Fig. 4f), in the WT females and CD163KO males in the piriform cortex (Fig. 4k) and only in males in amygdala and SN (Fig. 4p and Supplementary Fig. 6c). This highlights the relevance of the sex in the phagocytic immune response to α-syn pathology, with an apparent higher sensibility in males. Notably, the CD68 upregulation at 1 month was significantly reduced in the ipsilateral striatum and amygdala of PFF-CD163KO females compared to CD163KO males (Table 1 and Fig. 4h, r). Interestingly, in the striatum, both PFF-CD163KO males and females showed CD68+ cells of bigger size and shorter ramifications, suggestive of a hypertrophic/phagocytic phenotype in mice lacking CD163 (Fig. 4a). Therefore, females lacking CD163 showed a reduced early CD68-phagocytic response to α-syn pathology. The CD68 phagocytic activity was associated with α-syn aggregation, as supported by the significant positive correlations between MJF14 pathology and CD68 expression in the piriform cortex (Spearman *r* = 0.7549, *p* = 0.0007) and amygdala (*r* = 0.679, *p* = 0.001) at 1 month p.i.

At 6 months, striatal CD68 expression in PFF-injected animals decreased to those observed in the MONO groups (Supplementary Fig. 6f). At this time-point, quantification of Iba1+ microglia in SN (Fig. 4s–v) showed an ipsilateral microglia proliferation in PFF-CD163KO females (vs. ipsilateral in PFF-WT and MONO-CD163KO females, Fig. 4s, t, v). No significant differences in the number of Iba1+ microglia were found in the SN of the male groups or WT females (vs. MONO Fig. 4u, v). However, confirming a microglia response to neuronal α-syn pathology the percentage of homeostatic-surveilling Iba-1+ (Type A) was decreased in the ipsilateral side of these groups in favor to the more ramified and hypertrophic profiles (vs. contralateral, Supplementary Fig. 6j). Altogether, our observations suggest that CD163 promotes the early myeloid phagocytic response to α-syn neuronal pathology, and its absence in females results in a microglia proliferation in long term.

### Differential astroglial responses in α-syn PFF-injected CD163KO females

**Fig. 5:**
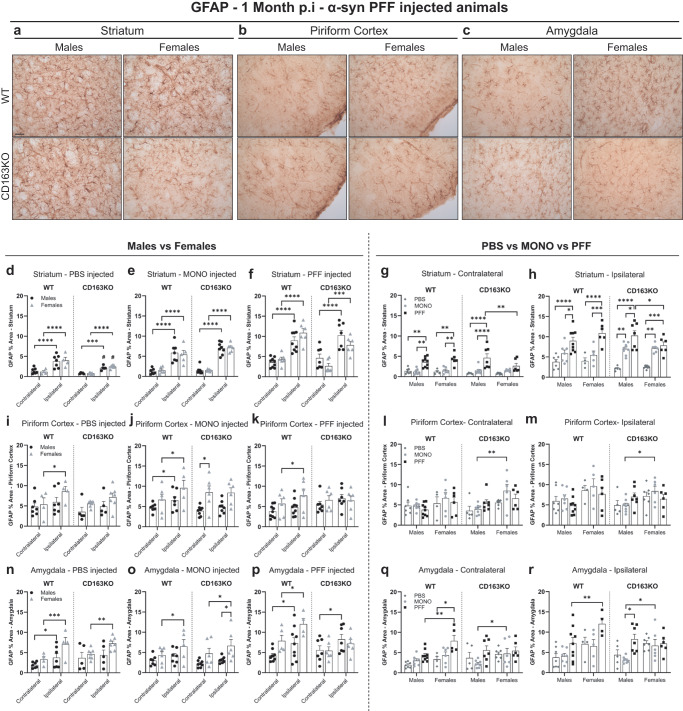
CD163KO females display dysfunctional astrocytic responses to α-syn PFF injection. **a** Representative images of GFAP immunostaining in α-syn PFF animals in the striatum, **b** piriform cortex and **c** amygdala at 1 month p.i. Scale: 50 μm applies to all. Bar graphs with individual points illustrate the percentage of area covered by GFAP+ staining after PBS, α-syn MONO and PFF injection in the **d**–**h** striatum, **i**–**m** piriform cortex and **n**–**r** amygdala at 1 month p.i. Values are mean ± SEM (*n* = 5–10). Statistics: two-way ANOVA followed by Sidak’s multiple comparison test. *P* values are given according to the number of symbols, e.g., *p < 0.05, **<0.01, ***<0.001, ****<0.0001. # different to the corresponding WT group.

Due to the relevant role of astrocytes in the CNS immune response^33^, we evaluated the GFAP expression in the different brain areas (Fig. 5 and Supplementary Fig. 7a–e). At 1 month, we found increased area covered by GFAP staining in the ipsilateral striatum of all PBS mice (vs. contralateral) (Fig. 5d), in the piriform cortex of CD163KO females (Fig. 5i) and in WT males and in all females in the amygdala (Fig. 5n). This suggests an early response of astrocytes to the surgery that appeared more relevant in females. In the α-syn MONO groups, we found increased GFAP expression in the ipsilateral striatum and SN in all mice (Fig. 5e and Supplementary Fig. 7b), in piriform cortex of WT (Fig. 5j) and only in females in the amygdala (Fig. 5o). Interestingly, MONO-CD163KO females showed higher GFAP expression than MONO-CD163KO males in the piriform cortex and amygdala (Fig. 5l, m, o, q). This again suggests an astroglia response to monomeric α-syn that is stronger in females.

At 1 month p.i. all PFF-mice showed ipsilateral (vs. contralateral) GFAP upregulation in the striatum and SN (Fig. 5f and Supplementary Fig. 7c), and in the amygdala except in CD163KO females (Fig. 5p). While only WT females showed ipsilateral GFAP upregulation in the piriform cortex (Fig. 5k). Notably, PFF-CD163KO females revealed lower levels of GFAP expression compared to CD163KO males in the ipsilateral striatum (Fig. 5h). The contralateral striatum of all PFF-animals, except the CD163KO females, also showed GFAP upregulation (vs. PBS and MONO), although to a lower extent than in the ipsilateral side (Fig. 5g). This bilateral GFAP upregulation was particularly seen in the amygdala of WT females, which was higher than in PFF-WT males (Table 1 and Fig. 5q-r). These observations highlight not only the sex relevance, with a stronger GFAP response in females, but also the anatomical differences in the astrocytic response to α-syn pathology. At 6 months p.i., GFAP expression levels in striatum and SN were similar between MONO and PFF animals and across hemispheres (Supplementary Fig. 7f, g). In conclusion, our results suggest that CD163 deletion leads to a damped astrocytic response to α-syn pathology in females early in the degenerative process in the model.

### Robust MHCII upregulation in α-syn PFF-injected males

**Fig. 6:**
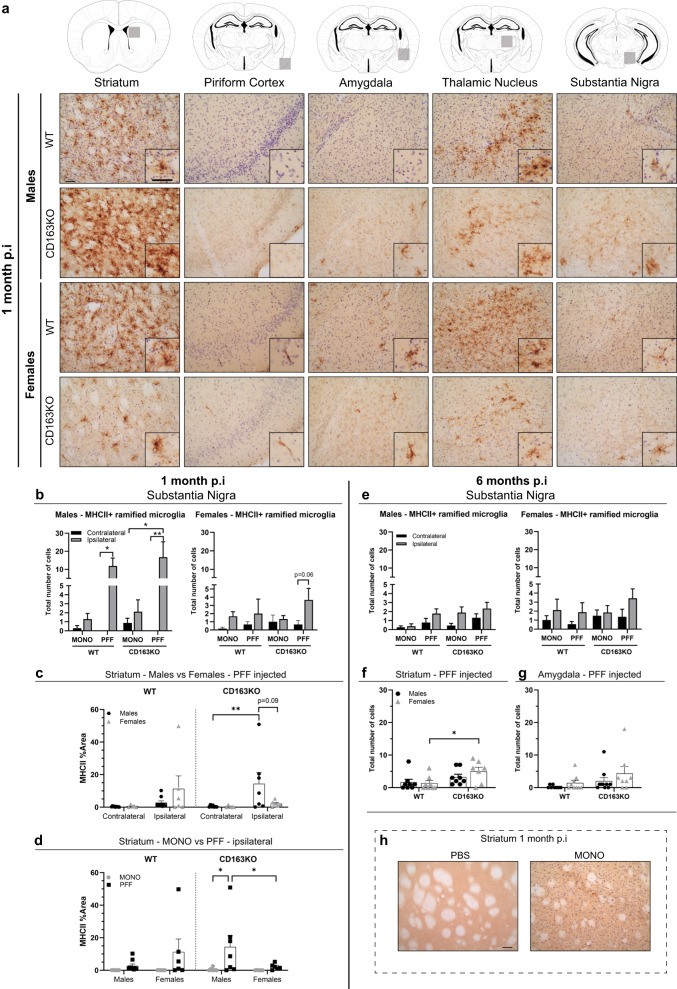
Expression of MHCII in the striatum and interconnected brain regions after α-syn PFF injection. **a** Representative images of MHCII immunostaining in α-syn PFF animals 1 month p.i. Areas marked by gray squares in the atlas sections above represent areas photographed. Bar graphs illustrate the number of MHCII+ cells in the SN at **b** 1 and **e** 6 months p.i. **c**, **d** Bar graphs with individual points show the percentage of area covered by MHCII in the striatum of at 1 month p.i. **f** Bar graph with individual points show the number of MHCII+ cells in the striatum and **g** amygdala at 6 months p.i. **h** Representative images of MHCII immunostaining in PBS and α-syn MONO-injected animals. Scale bar: 50 μm applies to all. Values are mean ± SEM (*n* = 6–10). Statistics: **c** Kruskal–Wallis test; **b**, **d**, **e** two-way ANOVA followed by Sidak’s multiple comparison test. *p < 0.05, **p < 0.01.

Expression of major histocompatibility complex II (MHCII) is a widely used marker of responsive microglia, and has been associated with α-syn pathology in PD models^5^ and in *postmortem* human PD brains^3^. As this model shows progressive bilateral α-syn pathology, both hemispheres were analyzed. Here, we found that MHCII expression—rarely found in microglia of the healthy brain (as confirmed in the PBS-injected animals (Fig. 6h))—was upregulated early in those regions with MJF14/pSer129+ α-syn pathology (Fig. 6a). In most brain regions, MHCII+ cells exhibited a ramified morphology (likely microglia), however, in the piriform cortex, we only observed numerous rod-shape MHCII+ cells, resembling perivascular macrophages (Fig. 6a). At 1 month, we observed substantially more MHCII+ ramified cells in the ipsilateral SN of all PFF-males (vs. contralateral), but mainly in PFF-CD163KO males that had higher MHCII expression than MONO-CD163KO males (Fig. 6b). Interestingly, fewer cells expressed MHCII in the female groups, and only the PFF-CD163KO females tend to show an ipsilateral MHCII upregulation (*p* = 0.06) (Fig. 6b). The robust ipsilateral MHCII upregulation in the CD163KO males, was also seen in the striatum, where MHCII expression was significantly higher than its contralateral (Fig. 6c), than MONO-CD163KO males and PFF-CD163KO females, which showed low levels of MHCII expression (Table 1 and Fig. 6d). After 6 months MHCII-expressing cells were rare in the SN of all animals (Fig. 6e). In the ipsilateral striatum, despite the very low number of MHCII+ cells, PFF-CD163KO females showed significantly more MHCII+ ramified cells than PFF-WT females (Fig. 6f). No differences were found in the ipsilateral amygdala (Fig. 6g). Therefore, males, predominantly CD163KO, respond strongly to α-syn pathology by upregulating MHCII early, while females do not. However, the absence of CD163 in females resulted in a late MHCII+ upregulation. This suggests that CD163 downmodulates the antigen presentation response to α-syn neuronal pathology, with CD163 in males dampening the early MHCII upregulation, while in females, CD163 prevents the long-term MHCII response.

### α-syn PFF CD163KO mice show a sex-dimorphic T cell response

**Fig. 7:**
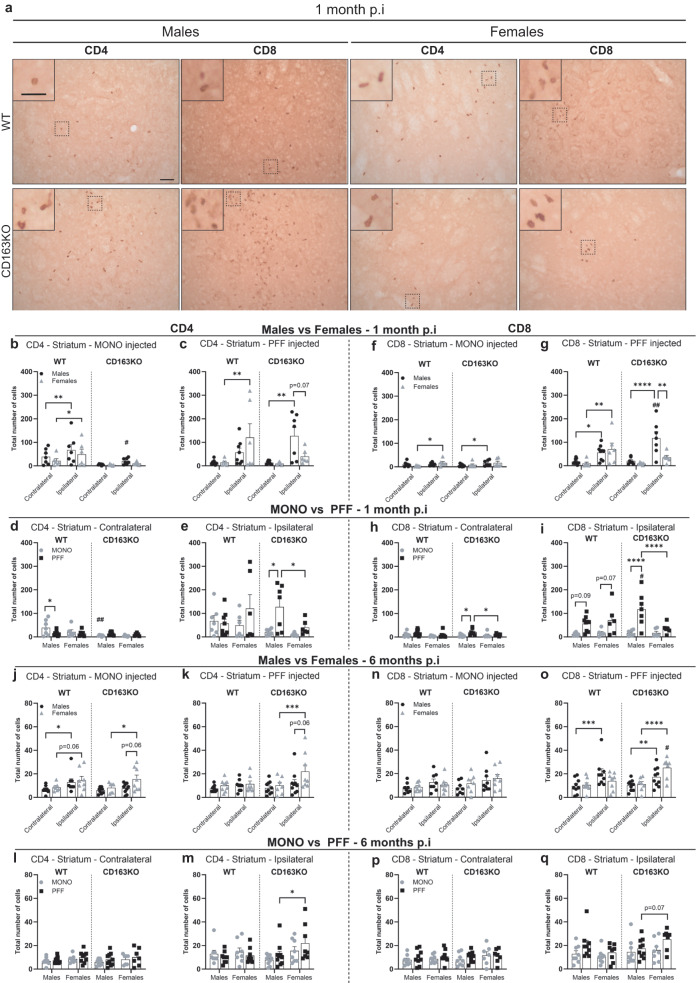
CD4-T and CD8-T cell infiltration in the Striatum after α-syn PFF injection. **a** Representative images of CD4 and CD8 immunostaining in α-syn PFF animals at 1 month p.i. Scale bars represent 50 and 25 μm for cropped images. Bar graphs with individual points show the total number of **b**–**e**, **j**–**m** CD4+ and **f**–**I**, **n**–**q** CD8+ cells after α-syn MONO and PFF injection at **b**–**i** 1 month and **j**–**q** 6 months p.i. Values are mean ± SEM (*n* = 6–10). Statistics: two-way ANOVA followed by Sidak’s multiple comparison test. *P* values are given according to the number of symbols, e.g., *p < 0.05, **<0.01, ***<0.001, ****<0.0001. # different to the corresponding WT group.

α-Syn aggregates and pathology have been shown to lead to MHC system upregulation that in turn will activate T cells^34,35^. In addition, since both CD4 and CD8 T cells have been observed in *postmortem* PD brains, we wondered if CD163 deletion would enhance T-cell brain infiltration in the striatum (Fig. 7). Interestingly, at 1 month p.i, we observed a bilateral CD4 T helper cell infiltration in MONO-WT animals, although significantly higher in the ipsilateral side (Fig. 7b, d, e). Yet, this was not seen in MONO-CD163KO animals (Fig. 7b, d). We also found low, although significant, ipsilateral CD8 T cell infiltration in MONO- WT females and CD163KO males (Fig. 7f).

At 1 month, α-syn PFF-mice showed significant CD4 T cells infiltration in the ipsilateral striatum in WT females and CD163KO males (vs. contralateral), but not PFF-CD163KO females, which had significantly fewer CD4 T cells than CD163KO males (Table 1 and Fig. 7c, e). Similarly, all α-syn PFF mice except CD163KO females showed significant CD8 T-cell infiltration in the ipsilateral striatum. Notably, CD8 T cell infiltration was enhanced in CD163KO males, which also showed significant, although lower, contralateral CD8 T cell infiltration (vs. MONO, Fig. 7f–i). Therefore, MONO α-syn WT mice showed a prompt bilateral CD4 T cell infiltration that was absent in CD163KO, while all PFF α-syn mice had ipsilateral CD4 and CD8 T cell infiltration, though of higher magnitude in CD163KO males and lower in CD163KO females.

After 6 months, despite the overall decrease in T cell numbers, all α-syn MONO mice, except CD163KO males, showed an ipsilateral infiltration of CD4 T cells in striatum (vs. contralateral) (Fig. 7j). Hence, the absence of CD163 avoided the short and long term CD4-T-cell infiltration induced by an excess of monomeric α-syn. However, in the α-syn PFF-mice, only CD163KO females had significantly more CD4 in the ipsilateral striatum than its contralateral and PFF-CD163KO males (Fig. 7k, m). At 6 months, CD8 infiltration was observed in the ipsilateral striatum of all PFF-males but also in CD163KO females, which showed more CD8+ cells than WT females and a similar trend vs. CD163KO males (*p* = 0.07) (Fig. 7o, q). Thus, PFF induced α-syn pathology in the absence of CD163 resulted in a long-term CD4 and CD8 T-cell infiltration in females, which was otherwise absent in WT females. In conclusion, CD163 modulates the innate immune responses and in turn adaptive immune responses to neuronal α-syn aggregation, by avoiding long-term T-cell infiltration in females.

### α-syn PFF-CD163KO females showed greater long-term dopaminergic loss

**Fig. 8:**
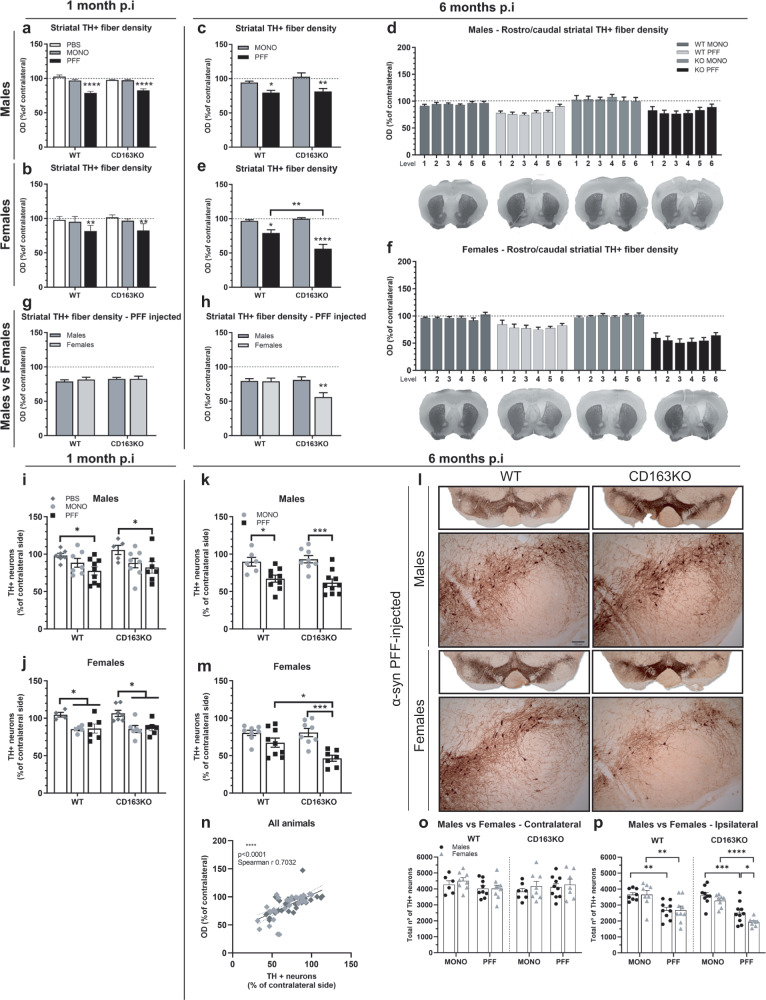
Striatal axonal density and stereological quantification of TH+ cell bodies in the substantia nigra. **a**–**h** Bar graphs illustrate the semi-quantitative measurement of TH+ axonal fiber density expressed as the percentage of the contralateral striatal side, post-PBS, α-syn MONO and PFF injection in the striatum. Average values of the 6 rostro-caudal striatal levels quantified are shown in **a** males and **b** females at 1 month p.i. Average and separate values of the 6 rostro-caudal striatal levels quantified are shown in **c**, **d** for males and **e**, **f** females at 6 months p.i, together with representative photos of one section at striatal level 0.38 from bregma in each group. Average values of the 6 rostro-caudal striatal levels quantified in α-syn PFF-injected animals are shown at **g** 1 month p.i and **b** 6 months p.i. **l** Low magnification photos show representative nigral sections immunostained for TH and higher magnification photos show the ipsilateral SN from WT and CD163KO male and female mice after α-syn PFF injection. **i**–**p** Stereological quantification of TH+ neurons in SN **i**–**j**, **k**, **m** as percentage of contralateral or **o**–**p** total number of neurons. Bar graphs with individual points illustrate the average number of surviving TH+ neurons in PBS, α-syn MONO and PFF-injected WT and CD163KO males and females at **i, j** 1 and **k**, **m** and α-syn MONO and PFF-injected animals at 6 months p.i; **n** correlation between the striatal TH+ axonal density and the number of TH + SN neurons (light gray symbols represent female and dark gray males). **o** Quantification of total number of TH+ neurons in the contralateral SN and in **n** ipsilateral SN. Scale bar for lower magnification photos represents 12.5 mm, and in higher magnification photos 100 μm as shown in (**l**). Values are mean ± SEM (*n* = 4–10). Statistics: two-way ANOVA followed by Sidak’s multiple comparison test. *p < 0.05, **<0.01, ***<0.001, ****<0.0001.

Given our observations on the alteration of α-syn pathology and sex-specific dysfunctional immune responses in CD163KO, we assessed whether such events led to distinct dopaminergic neurodegeneration (Fig. 8). Densitometry analysis of striatal TH+ fibers revealed an 18–21% decrease in the ipsilateral side in all α-syn PFF groups at 1 month (vs. PBS and MONO) (Fig. 8a, b), which remained after 6 months, in α-syn PFF males (Fig. 8c, d). However, PFF-CD163KO females showed a further reduction to 44%, resulting on significantly lower axonal TH-density than MONO-CD163KO, PFF-WT females and PFF-CD163KO males (Table 1 and Fig. 8e, f, h). No rostro/caudal pattern of dopaminergic denervation was seen (Fig. 8d, f).

Stereological quantification of SN TH+ cell bodies showed a decrease of TH+ neurons (as % of contralateral) in PFF-injected males and females (vs. PBS) but also MONO-injected females (Fig. 8i, j). However, after 6 months, PFF-WT and PFF-CD163KO males showed a 33% and 38% loss of dopaminergic neurons, respectively, in the ipsilateral SN (vs. MONO) (Fig. 8k). Notably, PFF-CD163KO females showed the biggest ipsilateral loss of dopaminergic neurons (54%) compared to MONO-CD163KO and PFF-WT females (Fig. 8m, l). No difference in the total TH+ numbers in the contralateral SN was seen (Fig. 8o); however, we found significant neuronal loss in the ipsilateral SN of all PFF-injected animals after 6 months. This was particularly enhanced in PFF-CD163KO females, which showed significantly fewer surviving neurons compared to MONO-CD163KO females, and PFF-CD163KO males (Table 1 and Fig. 8p). As expected, the striatal axonal TH+ density correlated significantly to the TH+ neuronal number in the SN (Fig. 8n). Moreover, the number of p62+ cells negatively correlated with the percentage of surviving TH+ neurons in SN of PFF animals (Supplementary Fig. 8b–h). This correlation was stronger in males (Supplementary Fig. 8c) and particularly driven by CD163 deletion (Supplementary Fig. 8e), suggesting that p62 accumulation is a significant factor in driving neurodegeneration and motor phenotype in CD163-deficient males. These findings suggest a neuroprotective role for the CD163 receptor in the progressive immune process triggered by neuronal α-syn aggregation in females.

### CD163 deletion alters the gene expression profile of macrophages and microglia after α-syn PFF injection

**Fig. 9:**
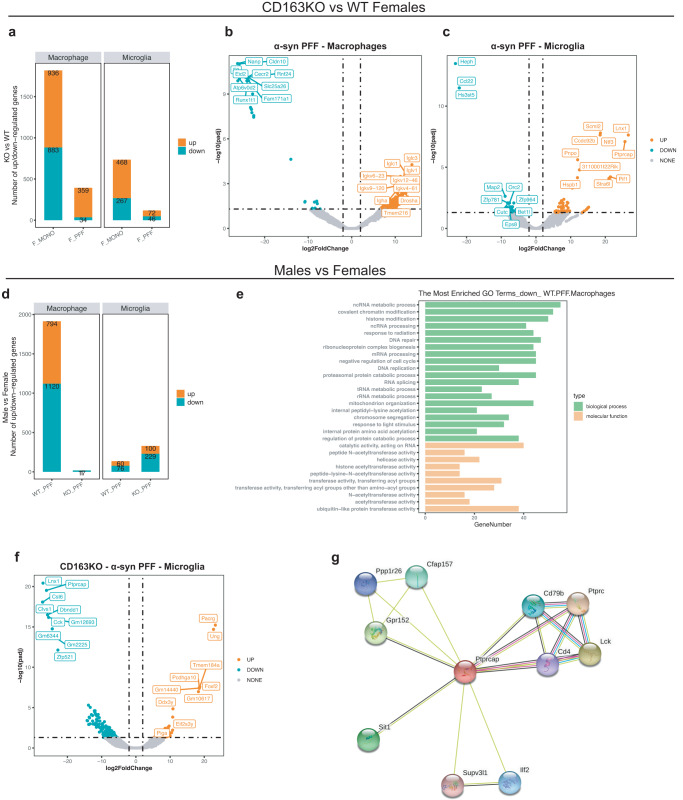
Microglial *Ptprcap* upregulation in CD163KO females and transcriptomic sex differences. **a** Bar graph showing the number of up/downregulated genes in CD163KO vs. WT females in Macrophage and Microglia populations 2 months p.i. **b** Volcano scatter-plots (-log10(padj) vs log2FoldChange) of DEGs (CD163KO vs. WT) in females-PFF Macrophages and **c** microglia. **d** Bar graph showing the number of up/downregulated genes in males vs. females (α-syn PFF groups) in Macrophage and Microglia populations 2 months p.i. **e** Gene Ontology (GO) analysis on the downregulated genes in WT-PFF (males vs. females) macrophages. **f** Volcano scatter-plot (-log10(padj) vs log2FoldChange) of DEGs (males vs. females) in CD163KO-PFF Microglia. **g** STRING protein–protein interaction network with focus on *Ptprcap* gene. Statistics: abs (logFC)>1, FDR < 0.05.

Based on the literature at 2 months p.i, the immune response related to the α-syn injection in the model has been resolved, α-syn pathology is significant, but no TH neuronal loss has occurred^36,37^. Therefore, to understand the impact of CD163 absence on the early immune response to α-syn neuronal pathology before cell death, we conducted SMART-seq2 at 2 months p.i. in the females (see Supplementary Fig. 9 for experiment design). We analyzed two FACS-sorted brain immune populations: microglia (CD45 low/intermediate/CD11b^+^), and macrophages (CD45 high/CD11b^+^), including brain-border-associated and infiltrated macrophages, neutrophils and NK cells (Fig. 9 and Supplementary Fig. 10).

Differentially expressed gene (DEGs) analysis revealed a reduced ability of the macrophage population in CD163KO females to respond to the α-syn neuronal pathology (PFF vs. MONO) compared to WT, which was paralleled by a differential response of the microglia population (Supplementary Fig. 10a). Few or no DEGs were shared between WT and CD163KO females, highlighting the significant impact of the CD163 deficiency on the immune response to the α-syn neuronal pathology (Supplementary Fig. 10b). In the macrophage population of PFF-WT females, upregulated genes were related to DNA damage response and regulation of catabolic processes (*Cecr2, Fzd6*), while genes associated with amino acid/protein metabolism (*Mpst, Akr1c12, Rsc1a1, Zfp354c*) and cytoskeleton organization (*Frmd5, Fhod3*) were downregulated (Supplementary Fig. 10c). PFF-CD163KO females showed a drastically reduced macrophage response, suggesting an impaired ability to mount an otherwise “normal” response to α-syn neuronal pathology. Enriched gene ontology (GO) functional annotations showed that the downregulated genes in the CD163KO females were involved in regulation of calcium homeostasis and metabolism (Supplementary Fig. 10d, g), known to play a central role in immune cell activation^38^. The microglia of the PFF-WT females showed upregulation of genes related to: peptide delivery to MHCI (*Lrmp*) and regulation of TLR4 surface expression (*Cnpy4*); while genes associated with: vesicle coating (*Clvs1*), DNA repair (*Mcm8, Trib2*) and metabolic mechanisms (*Got1l1, Acot11, Kyat1*) were downregulated (Supplementary Fig. 10e). Microglia from CD163KO females showed the most significant DEGs (lowest p value and highest fold change (PFF vs. MONO)). Including upregulation of: *Lnx1*, a gene related with ubiquitination and activation of proteosomal degradation mechanisms, *St8sia1*, associated with sphingolipid metabolism, and *Ptprcap*, encoding a key regulator of CD45-CD4 T-cell interaction through the TNFR1 pathway. Genes related to DNA damage response (*Atad5, Smad9*), apoptosis modulation (*Dab2ip*), cell migration (*Dnai3*) and cytoskeleton organization (*Slain1*) were downregulated (Supplementary Fig. 10f).

Since CD163KO females showed increased dopaminergic loss, we focused on comparing the response to PFF and MONO α-syn in CD163KO vs. WT females. PFF-CD163KO females (vs. PFF-WT) macrophages had significantly fewer DEG than those seen when comparing the MONO females’ groups (Fig. 9a) and were associated with downregulation of genes related to amino acid/protein metabolism (*Nanp*), cytoskeletal dynamics (*Fam171a1*), tight junctions and BBB integrity (*Cldn10*) (Fig. 9b). Microglia in PFF-CD163KO females was also affected (Fig. 9a), with downregulation of iron metabolism-related genes (*Heph*), and heparan sulfate biosynthesis mechanisms (*Hs3st5*). *Ccl22*, a chemokine related to T-cell migration was also downregulated suggesting that CD163 expression influences peripheral cell infiltration in females. GO analysis revealed that pathways affected by gene downregulation were limited to ubiquitin binding and DNA catalytic activity pathways (not shown). The most upregulated genes were: *Ntf3*, which encodes Neurotrophin3 a protein involved in neuronal survival and differentiation, and, once again, *Lnx1* and *Ptprcap* (Fig. 9c). Altogether, these results suggest that the early transcriptomic immune alterations seen in CD163KO females may contribute to their higher susceptibility to α-syn induced neurotoxicity.

To investigate sex differences in the immune response to α-syn neuronal pathology, we compared DEGs in PFF-males vs. PFF-females (Fig. 9d–f). Macrophage transcriptomes were very different between sexes in the PPF-WT group, where, in contrast to females, males presented many downregulated genes (1120, vs. female) mostly related to modulation of epigenetic machinery and DNA repair mechanisms (Fig. 9e), confirming a sex-dimorphism in the macrophage response to neuronal α-syn aggregation. Remarkably, this sex difference in DEGs was lost upon CD163 deletion, which mostly resulted in a few downregulated genes in male macrophages (Fig. 9d). However, the CD163 deletion resulted in more DEG in male microglia (vs. females), also compared to those in the WT group (Fig. 9d). The most highly upregulated gene found in PFF-CD163KO male microglia was *Pacrg*, which encodes the Parkin co-regulated protein, a protein found in Lewy bodies^39^ and related to aggresome formation and increased autophagy^40^. Once again, the most significant downregulated genes in CD163KO male microglia were *Lnx1* and *Ptprcap*, as they were highly upregulated in CD163KO females (vs. WT and MONO) (Fig. 9f). Interestingly, STRING analysis of the Ptprcap protein shows interaction with CD4, TNFR1 and TWEAK pathways (Fig. 9g). These results confirm the sex-dimorphic behavior of CD163 in the immune response to neuronal α-syn aggregation.

### CD163 deletion does not alter the in vitro response of macrophages to α-syn PFF

We have previously shown that the CD163KO does not drastically affect the in vitro immune response of macrophages^15^. To evaluate if this is also true for the α-syn PFF-induced response, we isolated bone-marrow-derived macrophages (BMDMs) from WT and CD163KO mice and stimulated them with either IL-10 or IFNγ for 24 h prior to α-syn PFF incubation (Supplementary Fig. 11). Expression of CXCL10, TNF and iNOS after BMDM priming with IFNγ, were significantly enhanced by α-syn PFF incubation in both WT and CD163KO males (Supplementary Fig. 11a, c, d) and females (Supplementary Fig. 11e, g, h). IL-1β expression, was significantly increased in all WT and CD163KO males BMDM primed with IFNγ and incubated with α-syn PFF (Supplementary Fig. 11b, f); but not in CD163KO females BMDM (Supplementary Fig. 11f, red bars), suggesting an irregular acute response to α-syn PFF. VCAM-1 and VEGFR1 (*Ftl-1*) mRNA were also measured, but no differences between groups were seen (not shown). Thus, the absence of CD163 does not drastically affect the ability of BMDM to acutely respond to extracellular α-syn PFF.

## Discussion

We investigated the role of the CD163 receptor in α-syn induced neurodegeneration and the associated immune response using the α-syn PFF PD model in CD163KO mice. Sex differences were considered given the reported sex dimorphism in the PD immune response, particularly related to CD163^41,42^. And the model progression was studied at 1, 2 and 6 months as previously^36,37,43^. α-syn PFF neuronal pathology resulted in early and sustained motor alterations in CD163KO males, but not in females. However, the PD model in CD163KO females showed a biphasic immune response that was diminished at 1 month but intensified after 6 months. This differential immune response was paralleled by an increased neuronal pathology at 6 months, which resulted in higher nigrostriatal dopaminergic degeneration. Transcriptomic analysis at 2 months p.i. revealed that CD163KO female macrophages lack the ability to respond to the α-syn neuronal pathology. This resulted in an altered microglial phenotype with upregulation of genes related to pro-inflammatory pathways, which may contribute to the enhanced neurodegeneration observed in CD163KO females. Overall, our data supports a sex-dimorphic role for CD163 in the immune response to neuronal α-syn-pathology, with CD163 downmodulating the innate and adaptive immune response, particularly in long term in females.

Intrastriatal injection of murine α-syn PFF induced aggregation and Ser129 phosphorylation of α-syn in the striatum and connected areas as before^35,44,45^. This was prominent in CD163KO animals and particularly enhanced in CD163KO females. Thus, CD163 deletion potentiates α-syn phosphorylation, increasing toxic α-syn aggregates^31,46^, especially in females. α-Syn aggregation and neuronal toxicity are associated with autophagic dysfunction and p62 accumulation^32^. Accordingly, PFF-injected mice showed α-syn pathology paralleled by p62 accumulation; and thus, although delayed compared to the other groups, it was higher in CD163KO females at 6 months. Thus, CD163 absence resulted in more prominent long-term autophagic changes in females. p62 colocalized with MJF14+ α-syn aggregates, and it was observed in SN dopaminergic neurons as seen in the rat PPF PD model^29^. Interestingly, we have prior in vitro observations showing that sCD163, produced during monocyte pro-inflammatory activation, increases the uptake of extracellular α-syn by monocytes and microglia, which should in turn clear it^26^. Our results here suggest that CD163 deficiency may compromise the ability of myeloid cells to properly respond to α-syn pathology, consequently promoting its pathological intraneuronal aggregation and p62 accumulation, particularly in females.

PD-immune response involves both brain and peripheral immune cells^2^. Accordingly, the α-syn-PFF PD model shows microgliosis^29,47^, presence of CD163+ cells in the brain^20^ and changes in peripheral immune cells^48–50^. We observed early CD68 phagocytic microglia activation, which coincided with MHCII upregulation. The early MHCII response was higher in the CD163KO males, suggesting an enhanced early adaptive immune activation in the absence of CD163, as confirmed by the higher T-cell infiltration. In contrast, PFF-CD163KO females lack this early immune response and also the GFAP upregulation seen in all other PFF-groups. However, after 6 months, when this immune response was resolved in most groups, it became relevant in the PFF-CD163KO females, which showed a significant unique SN microgliosis, higher MHCII expression, and persistent T cell infiltration in the striatum. This might ultimately contribute to higher neurodegeneration as T-cells seem to mediate dopaminergic loss in PD models^51,52^. In conclusion, myeloid cells in CD163KO females showed an inability to timely respond to early α-syn-associated pathology, which resulted in a delayed, but long-lasting neuroinflammation, involving both innate and adaptive cells.

Throughout the study, we observed site and sex-specific variations. The amygdala showed pronounced α-syn pathology and immune changes as seen before in the model and in patients^53,54^. Here, the amygdala had the most responsive astrocytes (reacting to mild conditions such as PBS or MONO), and was highly affected by the CD163 deletion, showing more bilateral pSer129 pathology in long term. However, microglia in the SN seemed particularly sensitive, with CD68 upregulation even after striatal PBS injections. This suggests an anatomically distinctive glia response to brain changes in agreement with data in healthy brains^55^ and in an α-syn PD model^56^. We also saw sex-specific changes (Table 1); in general, male mice (WT and CD163KO) tend to show stronger changes than females after PFF α-syn injections: with higher early upregulation of MHCII in SN, higher CD68 expression in the amygdala and long term CD8 T cell ipsilateral infiltration in the striatum. On the other hand, GFAP upregulation seemed more consistent in females, while MHCII expression was lower. In addition, PFF-WT females showed less robust p62 expression in SN after 6 months. This agrees with previous reports of a more “neuroprotective” profile of microglia in females^57^ vs. males, with a more inflammatory response and higher MHCII expressors^57,58^. Of relevance to the disease modeling, MONO α-syn injections were not innocuous and led to CD68 and GFAP upregulation, and CD4 T-cell infiltration in short and long terms in WT animals. This MONO response was affected by CD163 absence as no early T cell infiltration was seen in the CD163KO mice, and only females CD163KO showed it in the long term, which further supports a role for the CD163 in the adaptive response. The MONO α-syn injections resulted in phenotypic changes (vs. PBS mice) and a small decrease of TH+ cells in SN after 1 month, which might be associated with the early immune response observed. Thus, the use of monomeric α-syn as a control group constitutes a limitation of our study and should be considered when interpreting the data.

Further corroborating a differential immune response, our transcriptomic data showed that CD163KO leads to an impaired macrophage response and a differential microglia response to α-syn pathology in females; although specific anatomical immune differences might have been missed after using the full hemisphere. Most DEG in the female CD163KO macrophages were downregulated and involved processes such as: calcium homeostasis regulation^38^, and cytoskeleton organization, BBB integrity and immune cell transmigration, which could be associated with the early decreased T-cell infiltration in this group. PFF-WT female microglia showed upregulated genes coding for proteins previously related to α-syn/PD immune response (MHC system and TLR4)^2^. However, CD163KO females’ microglia downregulated *Ccl22*, a chemokine involved in the regulation of leukocyte migration^59^ that might also contribute to the decreased early T-cell infiltration in this group. Of particular interest CD163KO female microglia upregulated *Ptprcap*, a key regulator of CD45-CD4 T-cell interaction and activation^60^, also associated with activation of the TNFR1 and TWEAK pathways. *Ptprcap* upregulation has been previously shown in the midbrain of the α-syn viral-vector PD model^61^. Interestingly, sCD163 is suggested to act as a decoy receptor for TWEAK, regulating TWEAK-induced pro-inflammatory canonical NF-κB activation^62^. Notably, TWEAK has been associated with dopaminergic cell death and the pro-inflammatory activation of astrocytes in the MPTP PD model^63,64^; and TWEAK is increased in PD patients’ serum^64^. Therefore, upregulation of *Ptprcap* and possible TWEAK-mediated inflammation may also be involved in the higher susceptibility of CD163KO females to α-syn pathology. Although, further studies are required to investigate this possible connection.

The higher α-syn pathology and the differential immune response in the PFF-CD163KO females led to increased dopaminergic loss. CD163+ cells are found in the brain in rodent PD models^19,20,29^ and in PD *postmortem* brains^17^. We previously reported increased CD163+ monocytes numbers and in their CD163 expression levels in blood from patients with early PD^11^. Our data here supports a protective role for CD163 on myeloid cells. Accordingly, higher numbers of CD163 cells in the blood of RBD patients (prodromal PD) were associated with lower immune activation in the SN and better dopaminergic transmission in the putamen^27^. Intriguingly, here the CD163 neuroprotective role seems especially relevant in females. Remarkably, shedding of CD163 was reported to increase in the sera from female PD patients, but not in males, supporting a sex difference regarding the CD163 system/cells during PD^26^. Further supporting a sex dimorphism in the PD-associated immune response, blood monocytes transcriptome differs between male and female PD patients^10^. Our histological data and RNA-seq analysis of the innate cells in the WT animals further corroborate a sex-dimorphic immune response with potential implications for neuronal health. Sex differences in immune cells have been described before^57,65,66^. The observed CD163 behavior and dimorphic immune response might be related to the higher PD risk in males^67^ and the differential presentation of the disease among sexes^9^. CD163 deletion in females impaired the immune cells to properly respond to the initial neuronal α-syn pathology, resulting in a long-term increase of the MHCII-T-cell response, which ultimately influences the neurodegenerative outcome, while in males, CD163 absence increased the early MHCII-T-cell response and the motor defects associated with the neuronal α-syn pathology but not the cell death in SN.

Our behavioral analysis supports a relevant role for the immune system in the symptomatic disease presentation. While all α-syn injected mice show behavioral changes at 1 month (vs. PBS) this was more pronounced in the PFF CD163KO male. Moreover, only PFF CD163KO male showed asymmetry at 6 months p.i. suggesting a sex-related difference in the symptomatic manifestation of motor defects, influenced by the immune environment. Studies using males CD163KO showed increased phenotype and mortality in a model of intracerebral hemorrhage^16^; and enhanced disease severity and imbalanced CD4 Th1/Th2 responses in a model of rheumatoid arthritis^15^. Interestingly, despite the α-syn pathology and the dopaminergic degeneration seen in WT, we only observed long-term behavioral changes in the CD163KO mice, emphasizing the immune environment’s role in the motor phenotype particularly in males. We and others have previously reported that the α-syn PFF rodent PD models regardless of sex do not consistently exhibit robust motor defects, despite the nigral degeneration^29,44,68,69^. However, changes in the challenging beam in pesticide exposed PFF-PD model have been seen only in males, which was not related to bigger nigral TH loss or α-syn pathology, but to dopamine turnover and inflammatory changes^69^. A male-predominant affection has also been seen in other PD-relevant studies in mice^70,71^, and male MPTP-intoxicated monkeys showed higher inflammation markers than females^72^. This suggests that the immune environment significantly influences the motor phenotype, particularly in males, potentially mediated by soluble cytokines^73,74^ or peripheral cell infiltration, impacting microglia and neuronal function/firing^75^. Accordingly, sickness behavior was associated with peripheral inflammation responsible for sensorimotor defects^76^. In conclusion, the behavior impairments in CD163KO males could also be collectively due to the early MHCII upregulation and the T cell infiltration, the neuronal α-syn pathology and autophagic changes and the dopaminergic neurodegeneration.

Overall, our results suggest that, although CD163 deletion does not affect the acute in vitro monocytic response to fibrillar α-syn, it results in a differential early transcriptomic innate immune response in vivo. In males, CD163 deletion led to early increased T-cell infiltration and motor impairment, whereas in females, it increased the long-term innate response with subsequent T-cell infiltration and enhanced susceptibility to α-syn-induced neurodegeneration. Therefore, CD163 on myeloid cells appears to modulate the antigen-presenting response to α-syn-pathology, dampening T-cell infiltration in brain. This might be related to changes in the border-associated macrophages, which are CD163+ and recently proposed to be crucial mediators of the T-cell infiltration and neurodegeneration in another α-syn PD model^77^. Interestingly, CD163 expression is described in the disease-associated microglia (DAM) in AD^25^. According to scRNA-seq studies, CD163 is absent in homeostatic microglia^21–23^, thus its expression in the DAM indicates an ectopic expression due to a loss of the microglia identity during disease^78,79^, or, alternatively a de-differentiation of infiltrated CD163-macrophages. Notably, a recent scRNA-seq study PD patients’ brains also revealed CD163 expression in the DAM^24^. In AD, the subpopulation of CD163+ amyloid-responsive microglia was depleted in AD cases with APOE and TREM2 risk variants, suggesting a protective role^80^. Furthermore, CD163 upregulation has been associated with macrophages with neuroprotective capacity in an AD model^81^. Thus, it could be speculated that CD163 upregulation is associated with a protective compensatory mechanism exerted by myeloid cells occurring both in the brain and in the blood of PD patients^11,24^. In conclusion, we demonstrate that the α-syn-pathology associated innate immune responses involving CD163 expression differs between males and females, suggesting that the sex-dimorphism in PD may be due to immune differences. In addition, our data suggest that the CD163 upregulation in the myeloid compartment during neurodegeneration is a compensatory neuroprotective mechanism. Future studies may provide further molecular insights into the role of CD163 in brain diseases, and the mechanisms underpinning the gender dimorphism.

## Methods

### Animals

Adult male and female CD163KO (CD163^tm1.1(KOMP)Vlcg^) mice and WT (C57BL/6N) littermates (3–4 months old) (*n* = 179 total (*n* = 95 males (50 WT + 45 CD163KO); *n* = 84 females (43 WT + 41 CD163KO)) were used in this study^82^. Mice weighed 20–25 g (12 weeks old) at the time of the surgery and were housed a maximum 4–6 per cage, with ad libitum access to food and water, in a climate-controlled facility under a 12 h/12 h night/daylight cycle. All animal experiments were approved and performed under humane conditions in accordance with the ethical guidelines established by the Danish Animal Inspectorate and following EU legislation.

### Experimental design

To generate a PD model, murine α-syn PFF (10 µg, average size 33.87 nm), were used due to their higher seeding efficiency in mice compared to human α-syn PFF^83,84^. In parallel, PBS was used as an absolute control, and murine monomeric α-syn (MONO) was used as a control for α-syn pathology, based on its reported lack of seeding capacity in vitro^85^ and in vivo^86^. The number of animals per group used was based in previous published work with significant dopaminergic neuronal loss in SN with groups of *n* = 4–6^37,87^. Mice received unilateral intrastriatal injection of PBS, α-syn MONO, or α-syn PFF. For histological analysis, mice were sacrificed 1 (all three groups) and 6 months (only MONO and PFF) post-surgery (Supplementary Fig. 1). The 1-month group included: PBS (males WT *n* = 7 and CD163KO *n* = 5; females WT *n* = 4 and CD163KO *n* = 7), α-syn MONO (males WT *n* = 7 and CD163KO *n* = 8; females WT *n* = 6 and CD163KO *n* = 6), and α-syn PFF (males WT *n* = 9 and CD163KO *n* = 7; females WT *n* = 6 and CD163KO *n* = 6). While the 6-month group consists of α-syn MONO (males WT *n* = 8 and CD163KO *n* = 9; females WT *n* = 8 and CD163KO *n* = 8), and in the α-syn PFF (males WT *n* = 9 and CD163KO *n* = 10, females WT *n* = 9, and CD163KO *n* = 8). Motor behavior was assessed on the week before each end-point using the Challenging Beam Test and the Cylinder test. Mice were euthanized, perfused and brains processed for immunohistochemical staining. At times, not all animals of each group and time were used for each immunohistochemical marker to secure enough tissue to analyze multiple proteins. Figures show individual points, to inform on the number of animals analyzed for each marker or alternatively n number is given in figure legends.

For whole population RNA sequencing, a third and fourth group of mice (batch 1 and batch 2) received bilateral intrastriatal injection of α-syn MONO (males WT *n* = 7 and CD163KO *n* = 4*; females WT *n* = 7 and CD163KO *n* = 5), and α-syn PFF (males WT *n* = 7, and CD163KO *n* = 5; females WT *n* = 7 and CD163KO *n* = 5). Triplicates were used for sequencing. Mice were sacrificed 2 months post-surgery, their brains dissected and the immune cells (microglia and macrophages) isolated and FACS sorted for RNA purification and subsequent SMART-seq2 sequencing (Supplementary Fig. 9). *CD163KO-MONO males were excluded from the analysis due to inconsistencies in the technical replicates. For the in vitro experiment, BMDM were isolated from CD163KO and WT C57BL/6N male and female mice, aged 6–12 weeks (*n* = 5 per group). All animal experiments were approved and performed under humane conditions in accordance with the ethical guidelines established by the Danish Animal Inspectorate and following EU legislation.

### Protein purification and aggregation of mouse α-synuclein

Murine α-syn was recombinantly expressed in E. coli BL21 DE3 bacteria using the pRK172 plasmid encoding for mouse α-syn (kind gift from Prof. Virginia Lee (University of Pennsylvania)). The bacteria were pelleted by centrifugation and resuspended in buffer (50 mM Tris, 1 mM EDTA, 0.1 mM DTE, 0.1 mM PMSF, pH 7.0). The suspension was lyzed by sonication in a Branson Sonifier (Output control 7; dutycycle 50), and centrifuged at 20,000 × *g* for 30 min at 4 °C. The supernatant was boiled for 5 min and centrifuged at 48,000 × *g* for 30 min at 4 °C. Preceding ion exchange purification, the supernatant was dialyzed in 20 mM Tris pH 6.5, and filtered through a 45 µm filter. Murine α-syn was purified on POROS HQ 50 ion exchange chromatography with a continuous gradient of 0–100% 2 M NaCl in 20 mM Tris pH 6.5. The fractions with murine α-syn were isolated and further purified by reverse phase chromatography (C18) in order to remove nucleotides, and lipids bound to α-syn. This step also removes endotoxins (lipoglycans) from the sample (<0.5 EU/mg, confirmed using the Pierce^TM^ Chromogenic Endotoxin Quant Kit, ThermoScientific^TM^). The pure protein was dialyzed in 20 mM ammonium bicarbonate, lyophilized, and stored at –20 °C. In order to produce sterile PFF, mouse α-syn was solubilized in PBS (7.2 mM Na_2_HPO_4_, 2.8 mM NaH_2_PO_4_, 140 mM NaCl, pH 7.4) to a final concentration of 7 mg/mL, and further sterile filtered through a 0.22 µm sterile filter in a LAF bench. The solution was allowed to aggregate at 37 °C, 1000 RPM for 10 days. The insoluble α-syn PFF were isolated from unbound α-syn MONO by 20,000 × *g* centrifugation for 30 min at 20 °C and further resuspended in fresh sterile PBS. Protein concentration was determined by Bicinchoninic acid protein concentration assay (Pierce).

### Recombinant α-syn fibrils sonication and stereotaxic surgery

On the day of surgery, α-syn PFF was sonicated for 40 min (70 ms off/30 ms on, 30% output power, Branson 250 sonifier), and their hydrodynamic average length was determined prior and post-surgery by Dynamic Light Scattering (Wyatt DynaPro NanoStar), at 25 °C. Fibrils were of average = 33.87 nm (range: 25.90–41.10 nm), thus within the optimal size range of 29–49 nm previously reported^88^, and remained so during the surgery day (not shown). Mice were anesthetized (Medetomidine hydrochloride (1 mg/ml), Midazolam (5 mg/ml), and Fentanyl (0.05 mg/ml) in 0.9% NaCl for a final i/p. dose of 20 ml/kg) and placed in a stereotaxic apparatus (Stoeling). α-syn PFF or α-syn MONO (2 μl of 5 μg/μl, in sterile PBS) were unilaterally (or bilaterally for RNA analysis) injected into the striatum: AP +0.7/+0.9 mm (females and males respectively), ML ±2.0 mm (from bregma), and DV –3.0 mm (from dura)^89^. A s.c. antagonist solution (Flumazenil (0.1 mg/ml), Naloxone (0.4 mg/ml), and Antipamezol hydrochloride (5 mg/ml) in 0.9% NaCl for a final dose of 20 ml/kg) was used and fully awaken animals were placed back in their cages and i.p injected with Buprenorphine (0.3 mg/mL) for pain relief.

### Behavioral tests

The Challenging Beam Test was performed using a beam with four 25 cm frames of progressively decreasing width as described before^90^. Animals were trained for 2 days to transverse the beam from the widest (frame 1) to the narrowest section (frame 4). On the test day, a mesh grid was placed over the beam leaving approximately 1 cm space between the grid and the beam surface. Animals were video-recorded while crossing the grid-surface with five trials per animal. Frame 1 was excluded from the analysis due to variable reactions after first contact with the beam. Videotapes of each animal were rated for time to traverse the beam, number of errors, and number of steps for each of the trials and averaged per animal.

The spontaneous activity on the Cylinder test was assessed as formerly^90^. Spontaneous activity in a glass cylinder was videotaped for 3–7 min and a test was considered valid if the number of rears was equal or >20. To evaluate motor dexterity, the number of hindlimb steps was counted. For vertical activity, the number of rears was counted. Hindlimb steps were counted when the animal placed a paw in a different position on the cylinder floor, toward a frontal or backward movement. The percentage of total contralateral and ipsilateral steps, contralateral paw use, and total hindlimb steps were calculated.

### Perfusion and tissue processing

Animals were euthanized with an overdose of pentobarbital (400 mg/mL, 1:10 I.P.). Upon respiratory arrest, animals were transcardially perfused through the ascending aorta with ice-cold 0.9% NaCl solution, followed by 4% paraformaldehyde (PFA in 0.1 M phosphate buffer, pH 7.4). PFA-perfused brains were removed and post-fixed in the same 4% PFA solution for 2 h and transferred to a 25% sucrose solution (in 0.02 M NaPBS) overnight for cryoprotection. Brains were subsequently sliced into 40-μm-thick coronal sections using an HM 450 Slicer Microtome (Brock and Michelsen, Thermo Fisher Scientific) and separated in series of 6 for the Striatum and of 4 for the SN. Sections were stored at –20 °C in an anti-freeze solution.

### Immunohistochemistry with DAB detection and immunofluorescence

Immunohistochemical staining was done on free-floating brain sections as previously described^29^. Briefly, sections were blocked in appropriate 5% normal serum and then labeled overnight with anti-tyrosine hydroxylase (TH polyclonal, 1:750, Millipore, AB152), anti-aggregated α-syn MJFR-14-6-4-2 (MJF14 Monoclonal,1:25000, Abcam, Ab209538), anti-phosphorylated α-syn (pSer129 α-syn Monoclonal/D1R1R, 1:3000, Cell Signaling Technology, #23706), MHCII (Monoclonal/M5/114.15.2, 1:400, eBioscience, 14-5321), p62/SQSTM1 (Polyclonal, 1:2000, Nordic Biosite, 18420-1-AP), CD68 (Monoclonal/FA-11, 1:1000, BioRad, MCA1957GA), Iba-1 (Polyclonal, 1:1000, Wako Fujifilm, 019-19741), GFAP (Polyclonal, 1:5000, Abcam, ab7260), CD4 (Monoclonal/RM4-5, 1:500, BD Biosciences, #553043) or CD8 (Monoclonal/4SM15, 1:500, eBioscience, 14-0808-82) in 2.5% serum and 0.25% Triton-X-100 in KPBS at room temperature. Sections were washed with KPBS, pre-blocked for 10 min in 1% serum and 0.25% Triton-X-100, and incubated with the appropriate biotinylated secondary antibodies (1:200, Burlingame, CA Vector Laboratories) for 2 h. Afterward, avidin-biotin-peroxidase complex (ABS Elite, Vector Laboratories) was used and visualized using 3,3-diaminobenzidine (DAB) as a chromogen with 0.01–0.1% H_2_O_2_. Sections were mounted on chrome-alum gelatin-coated slides and coverslipped. For MHCII and Iba-1 staining, sections were counterstained with Cresyl violet (0.5% solution). Slides were analyzed using a Leica DMI600B brightfield microscope unless specified.

For immunofluorescence, free-floating sections were blocked in the appropriate 5% normal serum and then labeled overnight with anti-TH (polyclonal, 1:750, Millipore, AB152), anti-aggregated α-syn MJFR-14-6-4-2 (MJF14 Monoclonal, 1:10,000, Abcam, Ab209538), p62/SQSTM1 (C-terminus) (1:1000, Nordic Biosite, 318-GP62-C), GFAP (Polyclonal, 1:5000, Abcam, ab7260) and Iba-1 (Polyclonal, 1:1000, Wako Fujifilm, 019-19741). Sections were washed with KPBS and incubated for 2 h with species-specific fluorochrome-conjugated secondary antibodies (Alexa-Fluor 488 or 647, Invitrogen) plus DAPI (1:2000, Sigma-Aldrich A/S) for nuclear staining. Sections were mounted on chrome-alum gelatin-coated slides with Dako fluorescent mounting medium. Confocal images were obtained using an LSM 710 Meta Confocal microscope (Zeiss) with a 20×/0.8 M27 objective. Extraction of single z-frame and maximum intensity projections were performed with ImageJ (Fiji) software.

### Densitometric analysis of dopaminergic axonal striatal innervation

Striatal density of TH+ dopaminergic fibers was measured by analysis of optical density at 6 different rostro-caudal levels^89^: AP: +1.10; +0.62; +0.38; +0.14; –0.22; –0.58 mm relative to bregma. Immunostained sections were scanned using a densitometer (EPSON Perfection 3200 (1800 dpi resolution, grayscale), and the digital images acquired were analyzed with ImageJ (Fiji) software using a grayscale. The optical density in each section was corrected to an unspecific background measured in the corpus callosum of the same section. Data are shown as a percentage of the ipsilateral side vs. the contralateral side.

### Stereology and microscopic analysis

Unbiased stereological estimation of total TH+ cells in the SN was performed by an observer blind to the animal’s identity, using the optical fractionator principle and a Bright field Leica DM600B microscope with the NEWcast program (Visiopharm). A 1.25× low power objective (HCX PL Fluotar, Germany) was used to outline the SN based on its anatomical landmarks. Dopaminergic TH+ neurons were counted with a 40× objective (Leica, Germany) in a series of 1:4 sections covering the full SN (8–10 nigral sections per animal) from the rostral corner of the pars compacta to the caudal end of the pars reticulata (located between –2.70 and –3.88 mm from bregma^89^) with a counting frame of 56.89 μm × 42.66 μm and a step length of 110–165 as to count a minimum of 100 cells per SN and a CE < 0.1.

Using a Bright field Leica DM600B microscope, the total number of cells with aggregated MJF14+ α-syn, and p62+ structures were manually counted in SN sections (three equally distant per staining; between –2.46 and –3.64 mm from bregma)^89^. Total number of MHCII+ cells in the SN was manually counted in a series of 6 equally distant midbrain coronal sections (–2.46 to –3.88 mm from bregma), and CD4+ and CD8+ T cells were counted in striatal sections (three equally distant per staining; 1.18 and –0.22 mm from bregma). At 6 months p.i, MHCII+ cells in the striatum and amygdala were manually quantified in three equally distant coronal sections (1.18 and –0.22 mm from bregma).

Enhanced Focal Images (EFI) captured using the Upright Widefield Slide Scanner microscope (UWSSM) were used to analyze the area covered by immunostaining in two-three coronal sections stained for: phosphorylated α-syn (pSer129) in the striatum (between 1.18 and –0.22 mm from bregma), amygdala and piriform cortex (0.26 and –2.18 mm); MJF14+ aggregates in the amygdala and piriform cortex (0.26 and –2.18 mm); p62-expressing cells in the amygdala and piriform cortex (–0.26 and –2.18 mm); CD68 in the striatum (1.18 and –0.22 mm) and in the SN (–2.70 and –3.88 mm); GFAP in the striatum (1.18 and –0.22 mm) and in the SN (–2.70 and –3.88 mm), and MHCII in the striatum (1.18 and –0.22 mm from bregma), using ImageJ (Fiji).

Iba-1+ cells were counted with a 40× objective in 3 equally distant SN sections (–2.70 and –3.88 mm) aided by the VIS module (Visiopharm program). The counting frame (56.89 μm × 42.66 μm) was randomly located by the VIS module and methodically moved to sample the entire delineated region of the SN (step length of 75 μm) and all cells inside the counting frame were counted. A positive cell had a cresyl violet-stained nucleus covering an Iba1+ cell body. Four cellular profiles were defined as previously described^5^. The number of Iba-1+ cells per mm^2^ was calculated according to the area sampled in each section (calculated by the VIS module) and the total number of cells counted per section. The percentage of each morphological cell type was calculated as the total % of A, B, C, and D types in each section and averaged per animal.

### Brain cell isolation and fluorescence-activated cell sorting for RNA isolation

Animals were transcardially perfused through the ascending aorta with ice-cold PBS, brains were extracted and cells isolated using the Adult Brain Dissociation Kit (Miltenyi Biotec) in accordance with the manufacturer’s protocol. Briefly, brains were cut into approximately 0.5 cm pieces and transferred into gentleMACS C Tubes containing the provided enzymes. Tissue was dissociated using the gentleMACS Octo Dissociator with Heaters for 30 min in the appropriate program. Homogenates were filtered through 70 μm cell strainers and the cell suspension was centrifuged at 300 × *g*, 10 min at 4 °C. Cells were resuspended in a Debris Removal Solution (in DPBS containing CaCl_2_, MgCl_2_, 1 g/L D-Glucose, and 36 mg/L Pyruvate, Gibco) and centrifuged at 4 °C and 3000 × *g* for 10 min. Gradient centrifugation formed three phases and the top two layers containing myelin debris were removed. Cells were washed with 1x PBS and counted in the MOXI Z Mini Automated cell counter (ORFLO).

Freshly isolated brain cells were blocked with CD16/CD32 (clone 2.4G2, Mouse BD Fc block, BD Pharming) and 10% goat serum in PBS for 10 min at 4 °C. Single-cell suspensions were incubated in darkness at 4 °C for 30 min with CD11b antibody conjugated to BV421 dye (50 μg/mL, clone M1/70, BioLegend) and CD45 antibody conjugated to APC dye (0.2 mg/mL, clone 30-F11, BioLegend). Cells were washed with PBS and 0.5% BSA, centrifuged at 4 °C and 400 × g for 5 min, and resuspended in cold PBS. Propidium iodide (PI) was added to the cell suspension for the identification of dead cells. Microglia and macrophage populations were sorted into separate tubes containing PBS, in the FACSAria III high-speed cell sorter (BD Biosciences, San Jose, CA) according to CD11b (405 nm laser and 450/40 bandpass filter) and CD45 (633 nm laser and 660/20 bandpass filter) Identification of total cells and exclusion of debris was performed based on SSC-A vs. FSC-A, followed by doublet exclusion by FSC-A vs. FSC-H. Exclusion of dead cells was done based on PI-A vs. CD11b-BV421-A. Microglia (CD11b^+^/CD45^low/intermediate^) and macrophages (brain-border-associated and infiltrated, neutrophils and NK cells, CD11b^+^/CD45^high^) were identified based on CD45-APC-A vs. CD11b-BV421-A (Supplementary Fig. 9). Once sorted, microglia (200,000–500,000 cells) and macrophage (10,000–30,000 cells) cell suspensions (>90% purity after sorting) were centrifuged at 4 °C and 400 × g for 5 min, resuspended in RLT Plus Lysis buffer (QIAGEN, Germany) with 1% β-Mercaptoethanol and vortexed for 1 min for cell lysis. The solution was further homogenized using a 1 mL syringe and a 21gag needle. Total RNA was extracted using RNeasy® Mini Kit (QIAGEN) according to the manufacturer’s protocol.

### SMART-Seq2, gene mapping, expression and analysis

Total purified RNA was sent to BGI Hong Kong, where Switching Mechanism at 5’ End of RNA Template (SMART-seq2) sequencing service was requested. Samples were tested for quality control using Agilent 2100 Bioanalyzer (Agilent RNA 6000 Nano Kit). RNA integrity (RNI) >7 was considered suitable for sequencing. Gene expression analysis was performed through full population RNA sequencing using the SMART-seq2 method according to BGI’s standard methodology.

Reads mapped to rRNA were removed and raw data was obtained. The sequencing reads that contained low-quality, adaptor-polluted, and high content of unknown base (*N*) reads processed were removed before downstream analyses. Clean reads were mapped to reference using Bowtie2^91^, and then gene expression level was calculated for each sample with RSEM^92^, a software package for estimating gene and isoform expression levels from RNA-Seq data. Based on the gene expression level, we identified the differentially expressed genes (DEGs) between α-syn PFF vs. MONO, male vs female, and WT vs. CD163KO groups. We use DESeq2 package of R^93^ to detect the DEGs, determining at a cutoff of FDR corrected *p* value ≤0.05 and |log2(fold change)| ≥ 1 as DEGs.

Principal component analysis (PCA) was performed on the Macrophage and Microglia population respectively based on the rlog of dds result from DESeq2 and draw the diagrams with the ggplot2 function of R. Batch effect was corrected and three outlier samples were removed according to the results of PCA diagrams (Supplementary Fig. 12). With DEGs, we performed Gene Ontology (GO) and KEGG pathway classification and functional enrichment using the ClusterProfiler package of R^94^. GO has three ontologies: molecular biological function, cellular component, and biological process. We performed biological process and molecular biological function enrichment respectively, showed when relevant.

### In vitro treatment of bone-marrow-derived macrophages and gene expression analysis

BMDMs were prepared by flushing the femur and tibia of CD163KO or WT mice, aged 6-12 weeks, with RPMI-1640. Flushed bone marrow was filtered through a 70-μm cell strainer (Corning®), and 6 × 10^6^ cells were plated in uncoated petri dishes (Greiner Bio-one) in RPMI-1640 with 10% fetal bovine serum (FBS, Biowest), penicillin and streptomycin and 2mM L-Glutamine (Gibco), supplemented with 10% L929 conditioned medium. Cells were supplemented with RPMI with 10% FBS, P/S, L-glut, and 10% L929 medium on day 4, and on day 7 the cells were harvested with TrypLE (Gibco) and plated in 24 well plates for downstream assays. Plated BMDMs were incubated with 10 ng/ml IL-10 or IFNγ (Peprotech) for 24 h and subsequently stimulated with PBS control or 1 μM murine α-syn PFF for 6 h, after which the cells were lyzed in TRIdity G^TM^ (Applichem). RNA was purified using the Direct-zol RNA kit (Zymogen), according to the manufacturer’s protocol. The quantity and quality of the RNA was measured using a Nanodrop ONE (Thermo Fischer Scientific), and cDNA was prepared using the High-Capacity cDNA Reverse Transcription Kit (Applied Biosystems). qPCR was performed using the KAPA SYBR® fast low ROX (Sigma-Aldrich), 5 ng RNA, and 0,4 μM combined forward/reverse primer on Applied Biosystems 7500 fast qPCR machine, and peptidylprolyl isomerase A was used as reference gene to calculate the relative gene expression (ΔCt). See primers used in Supplementary Table 1.

### Statistical analysis

All analyses were done by researchers blind to the group’s identity. All statistical analyses were performed using GraphPad Prism v.10.0.1 (GraphPad Software, California, USA). Gaussian Normality was first assessed using normality distribution with a Shapiro–Wilk normality test followed by two-way ANOVA and, when appropriate, by a post-hoc Sidak’s multiple comparisons test. Outliers were analyzed using ROUT method (*Q* = 5%). Bonferroni corrections were applied when appropriate. Alpha was set at 5%.

### Supplementary information

Supplementary Info

Related Manuscript File

## Data Availability

The authors confirm that the data supporting the findings of this study are available within the article and its supplementary material (available online), or otherwise are available from the corresponding author, upon reasonable request. High-throughput RNA sequence data have been deposited to the Gene Expression Omnibus (Accession number GSE243536).
