# Supplementary material for: Sex-dimorphic neuroprotective effect of CD163 in an α-synuclein mouse model of Parkinson’s disease
Source: NPJ Parkinsons Dis. 2023 Dec 13;9:164. doi: 10.1038/s41531-023-00606-w (PMC10719342; doi:10.1038/s41531-023-00606-w)
Supplement: Supplementary file 1 — Supplementary Info [file 41531_2023_606_MOESM1_ESM.pdf]

Supplementary information

Sex-dimorphic neuroprotective effect of CD163 in an α-synuclein mouse model of Parkinson's disease

Sara A. Ferreira[1,2], Conghui Li[4], Ida H. Klæstrup[1,2], Zagorka Vitic[1,2], Rikke K. Rasmussen[1], Asger Kirkegaard[1,2], Gitte U. Toft[1,2], Cristine Betzer[1,2], Pia Svendsen[1,3], Poul H. Jensen[1,2], Yonglun Luo[1,2,4], Anders Etzerodt[1], Søren K. Moestrup[1,3] and Marina Romero-Ramos*[1,2]

Supplementary Table 1. Primers used for gene expression analysis of bone marrow-derived macrophages

Target	GeneID	Sequence (forward/reverse)
Ppia	NM_013494.4	5′-ATGGTCAACCCCACCGTG-3'
		5'-TTCTTGCTGTCTTTGGAACTTTGTC-3'
Cxcl10	NM_021274.2	5'-GGGCCATAGGGAAGCTTGAA-3'
		5'-GGATTCAGACATCTCTGCTCATCA-3'
Il1b	NM_008361.3	5'-TGGCAACTGTTCCTGAACTCA-3'
		5'-GGGTCCGTCAACTTCAAAGAAC-3'
Tnf	NM_013693.3	5'-GGGTGATCGGTCCCCAAA-3'
		5'-TGAGGGTCTGGGCCATAGAA-3'
Nos2	NM_010927	5'-GCCACCAACAATGGCAACAT-3'
		5'-TCGATGCACAACTGGGTGAA-3'
Flt1	NM_010228	5'-GAGGAGGATGAGGGTGTCTATAGGT-3'
		5'-GTGATCAGCTCCAGGTTTGACTT-3'
Vcam1	NM_011693	5'-CTCTTACCTGTGCGCTGTGA-3'
		5'-CTTCAGGGAATGAGTAGACCTCC-3'

Supplementary Figures

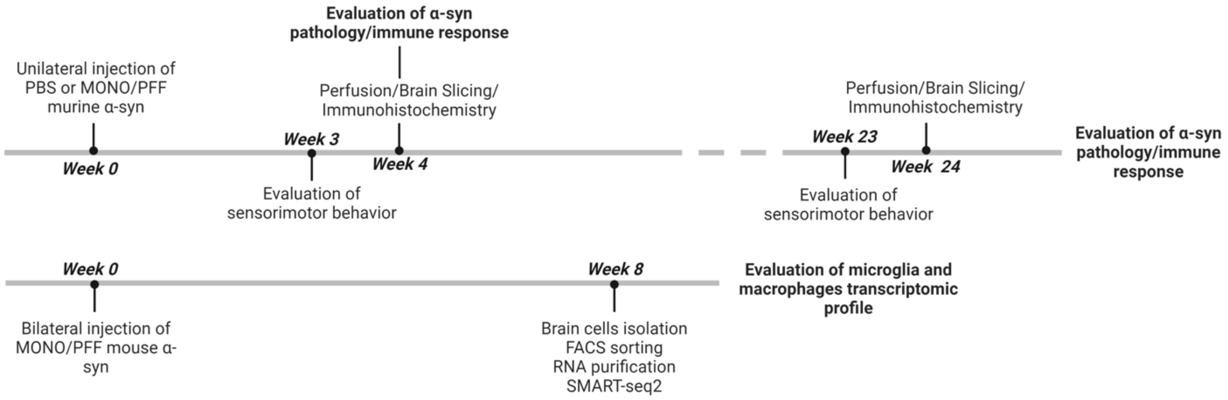

Supplementary Fig. 1 Experimental study design. Graphic Illustration of experimental study design for histology study and RNA sequencing study. Created with Biorender.com

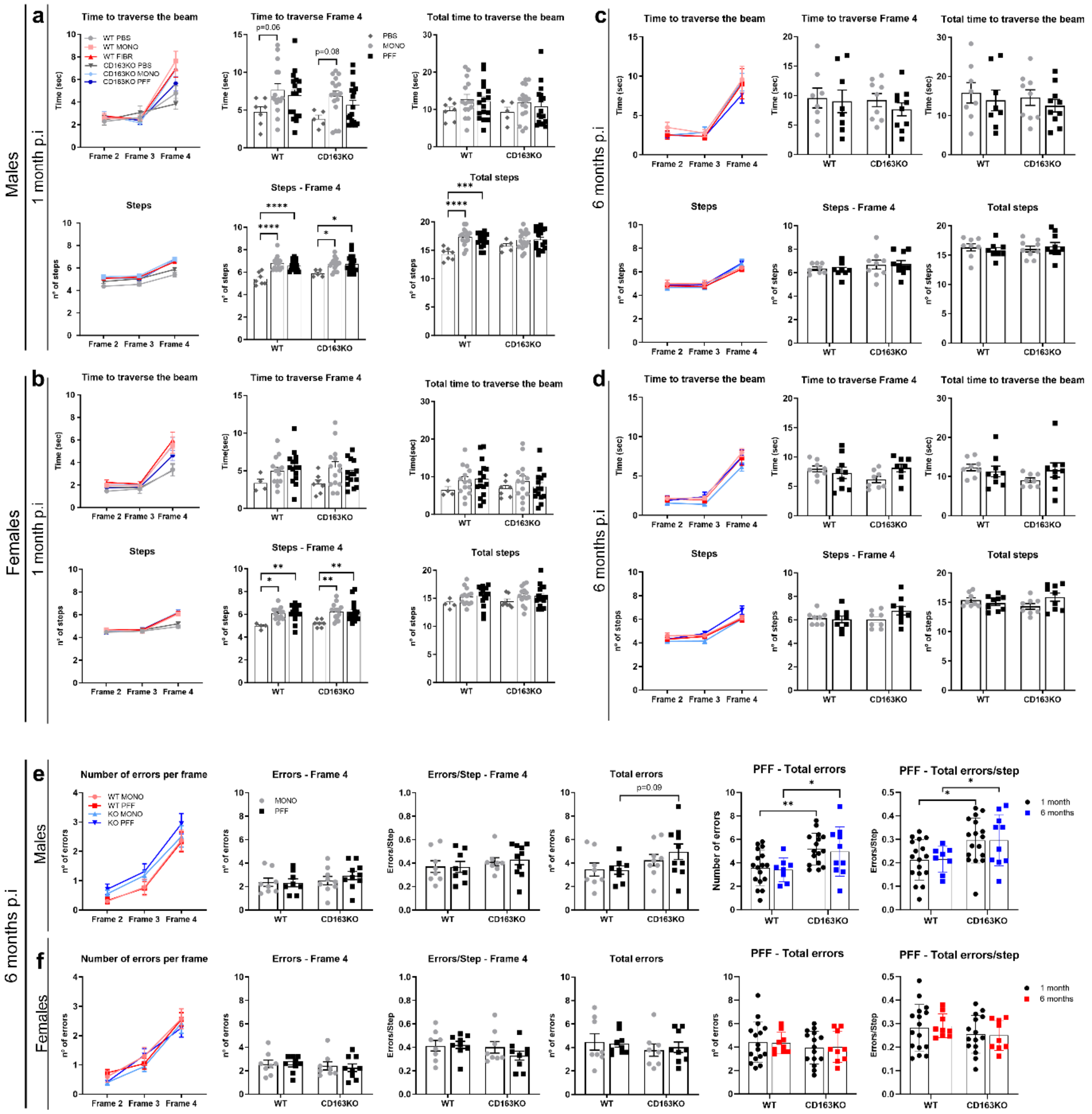

Supplementary Fig. 2 Assessment of motor performance on the Challenging Beam test. Line and bar graphs with individual values illustrate the quantitative measurement of the number of time and steps to traverse the beam, time and steps to traverse Frame 4 and total time/ total steps to traverse the beam (combination of Frame 2, 3 and 4) in the Challenging Beam test at **a-b)** 1-month post- PBS or α-syn MONO/PFF unilateral injection in the Striatum and **c-d)** 6-months post-α-syn MONO/PFF injection in **a, c)** males and **b, d)** females. Line and bar graphs with individual values illustrate the quantitative measurement of the number of errors per frame, number of errors and errors/step in Frame 4 and total number of errors (combination of Frame 2, 3 and 4) in the Challenging Beam test, 6-months post-α-syn MONO/PFF unilateral injection in the Striatum of **e)** males and **f)** females. Values are Mean±SEM (n=14-17 for behavior at 1-month; n=8-10 for behavior at 6-months). Statistics: Two-way ANOVA followed by Sidak's multiple comparison test.

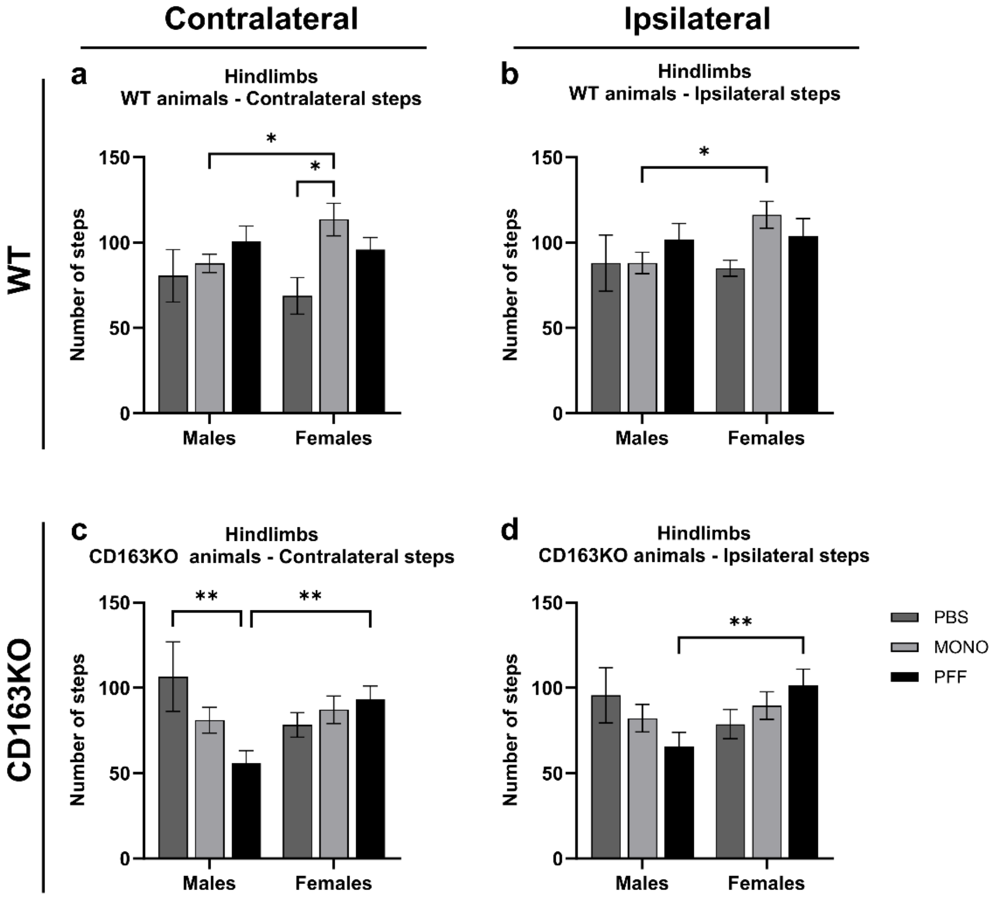

Supplementary Fig.3 Assessment of motor performance on the Cylinder test 1 month post-injection. Bar graphs represent the number of **a,c)** contralateral and **b,d)** ipsilateral hindlimb steps in the cylinder in **a-b)** WT and **c-d)** CD163KO males and females. Values are Mean±SEM (n=4-7 (PBS group), 14-17 (MONO/PFF groups) Statistics: Two-way ANOVA followed by Sidak's multiple comparison test. *p<0.05, **<0.01, ***<0.001, ****<0.0001.

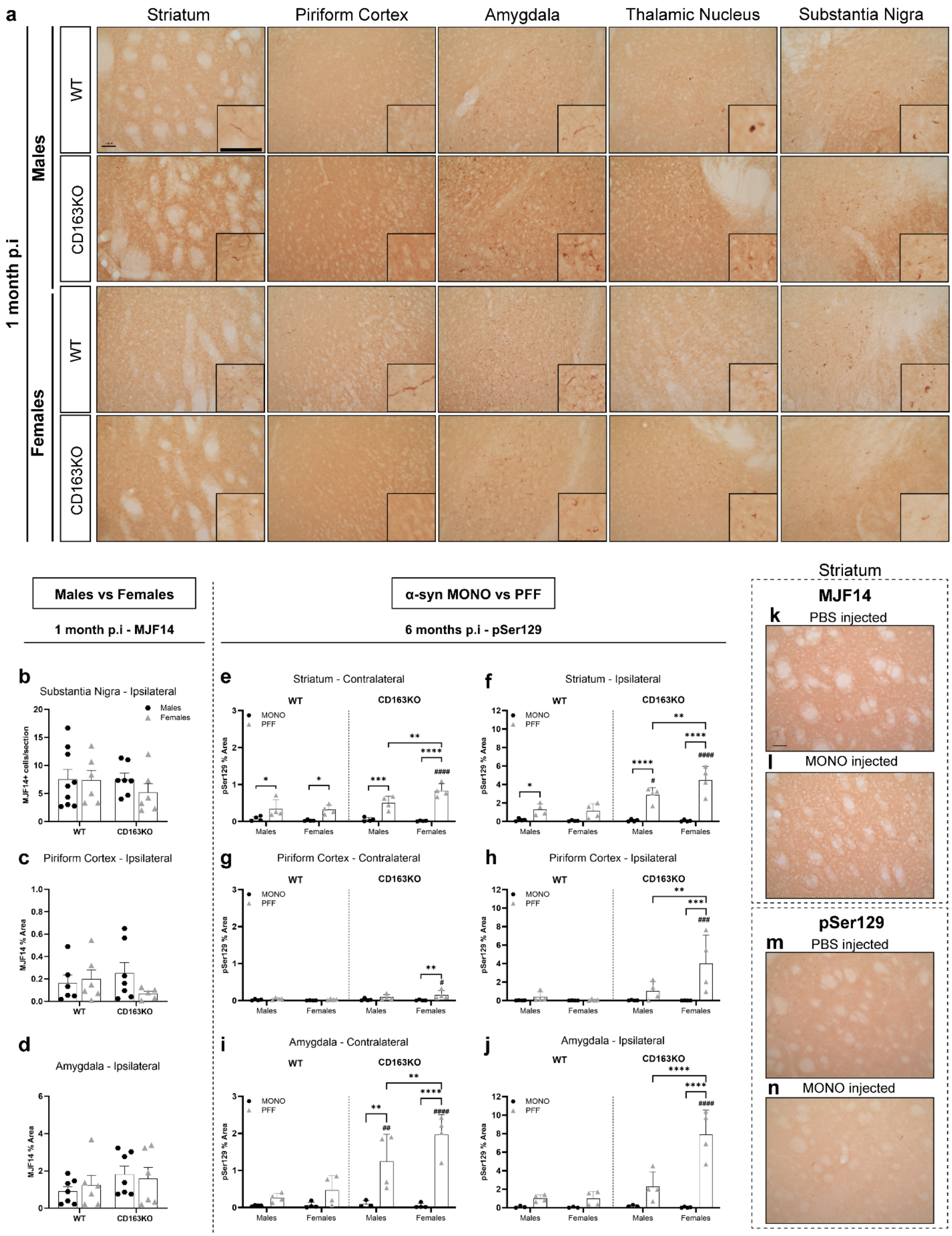

Supplementary Fig.4 Spreading of pathological α-syn through the basal ganglia and interconnected regions. a) Representative images of MJF14 immunostaining in α-syn PFF-injected animals at 1-month p.i. **b)** Bar graphs with average and individual points represent the number of MJF14+ cell aggregates in the ipsilateral substantia nigra, and the percentage of area covered by MJF14+ staining at 1-month post-α-syn-PFF injection in the **c)** ipsilateral piriform cortex and **d)** amygdala. **e-f)** Bar graphs with individual values represent the percentage of area covered by phosphorylated (pSer129) α-syn in the contralateral and ipsilateral striatum, **g-h)** piriform

cortex and **i-j)** amygdala 6-month post-α-syn injection. Representative striatal images of **k-l)** MJF14 and **m-n)** pSer129 immunostaining in PBS and α-syn MONO-injected animals at 1-month p.i. Values are Mean±SEM (n=6-9 for MJF14) and (n=4 for pSer129). Statistics: Two-way ANOVA followed by Sidak's multiple comparison test. P values are given according to the number of symbols e.g. *p<0.05, **<0.01, ***<0.001, ****<0.0001. The symbol # indicates different to the corresponding WT group. Scale bar: 50μm applies to all.

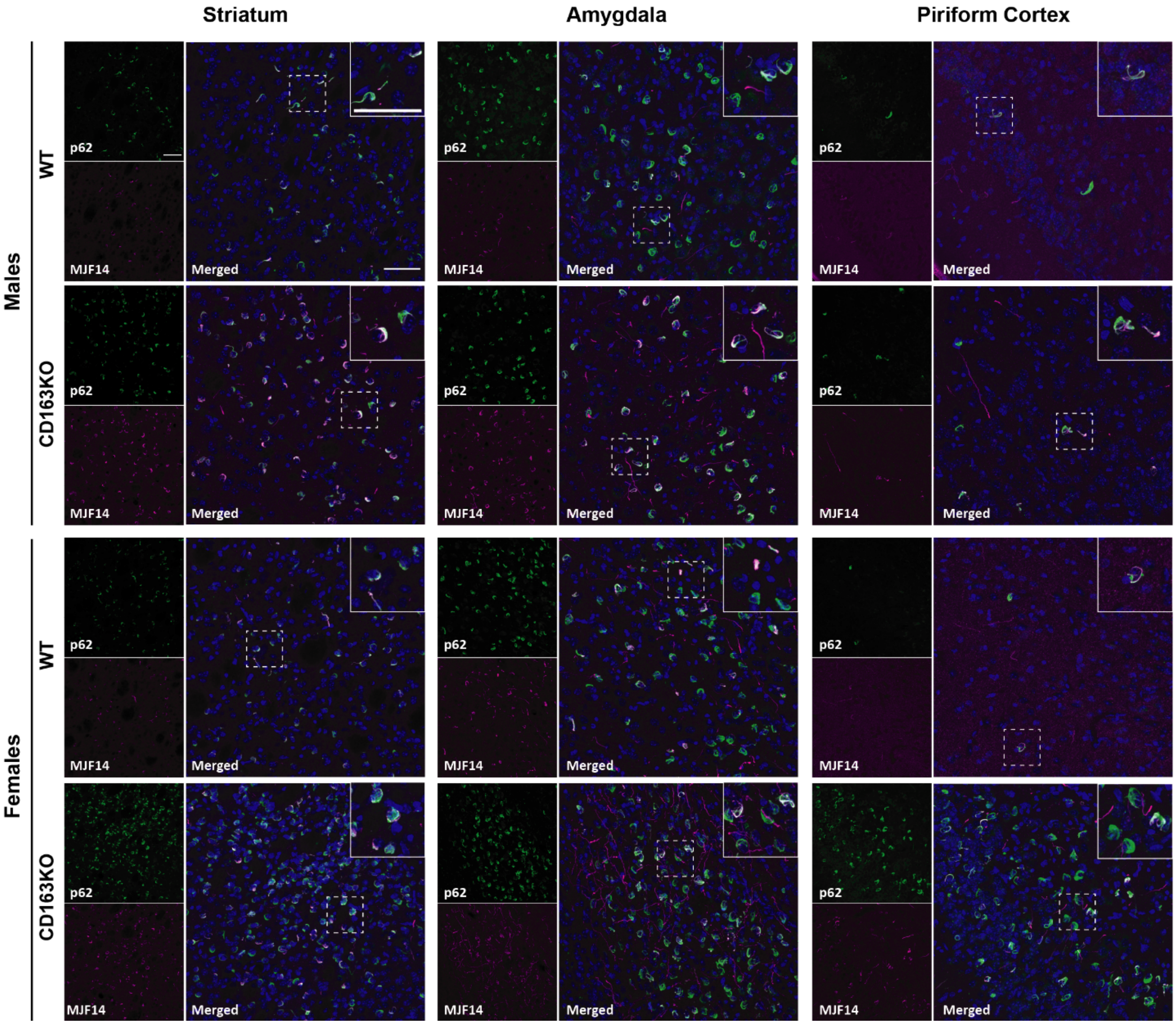

Supplementary Fig.5 Aggregated α-syn is associated with p62 accumulation. Representative confocal images of MJF14 (magenta) and p62 (green) and merged photo (with nuclear DAPI (blue)) in the ipsilateral striatum, amygdala and piriform cortex of α-syn PFF animals 6 months p.i. Squares with white dashed lines delineate the cropped region of the amplified images (upper right corner). Scale bar: 50 µm.

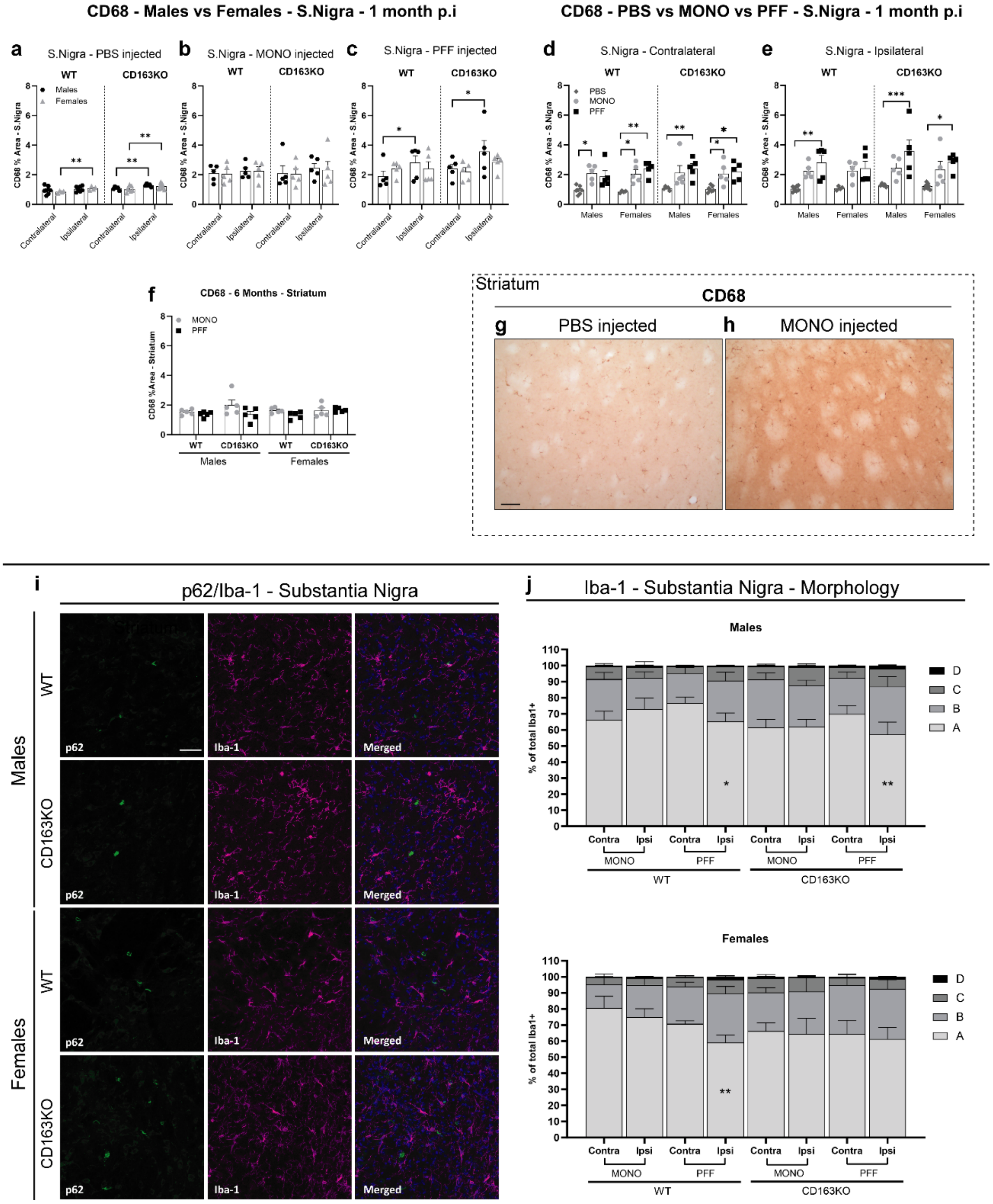

Supplementary Fig.6 CD68 and Iba-1 expression after α-syn injection. Bar graphs with individual values represent the percentage of area covered by CD68 staining in the **d)** contralateral and **e)** ipsilateral SN 1 month post- **a)** PBS **b)** α-syn MONO and **c)** α-syn PFF injection; and in the **f)** ipsilateral striatum 6 months post- α-syn MONO/PFF injection. Representative striatal images of CD68 immunostaining in **g)** PBS and **h)** α-syn MONO-injected animals at 1-month p.i. Representative confocal images of p62 (green) labeling with **i)** Iba-1 (magenta) at 6 months p.i, displaying individual channels and merged/composite images with (DAPI (blue)) in the ipsilateral SN of α-syn PFF animals. Scale bar: 50 µm applies to all. **j)** Stacked bar graphs illustrate the percentage of total Iba-1 positive cell subtypes (A, B, C and D) in the contralateral and ipsilateral SN, 6 months after α-syn MONO and PFF-injection in Males (upper graph) and Females (lower graph). Values are Mean±SEM (n=6-9 for CD68) and (n=5 for Iba-1). Statistics: a-f) Two-way ANOVA followed by Sidak's multiple comparison test. *$p<0.05$,

<0.01, *<0.001. **j)**: Two-way ANOVA (side and subtype) followed by Sidak's multiple comparison test. *p<0.05, **<0.01 (compared to contralateral in the same group and subtype).

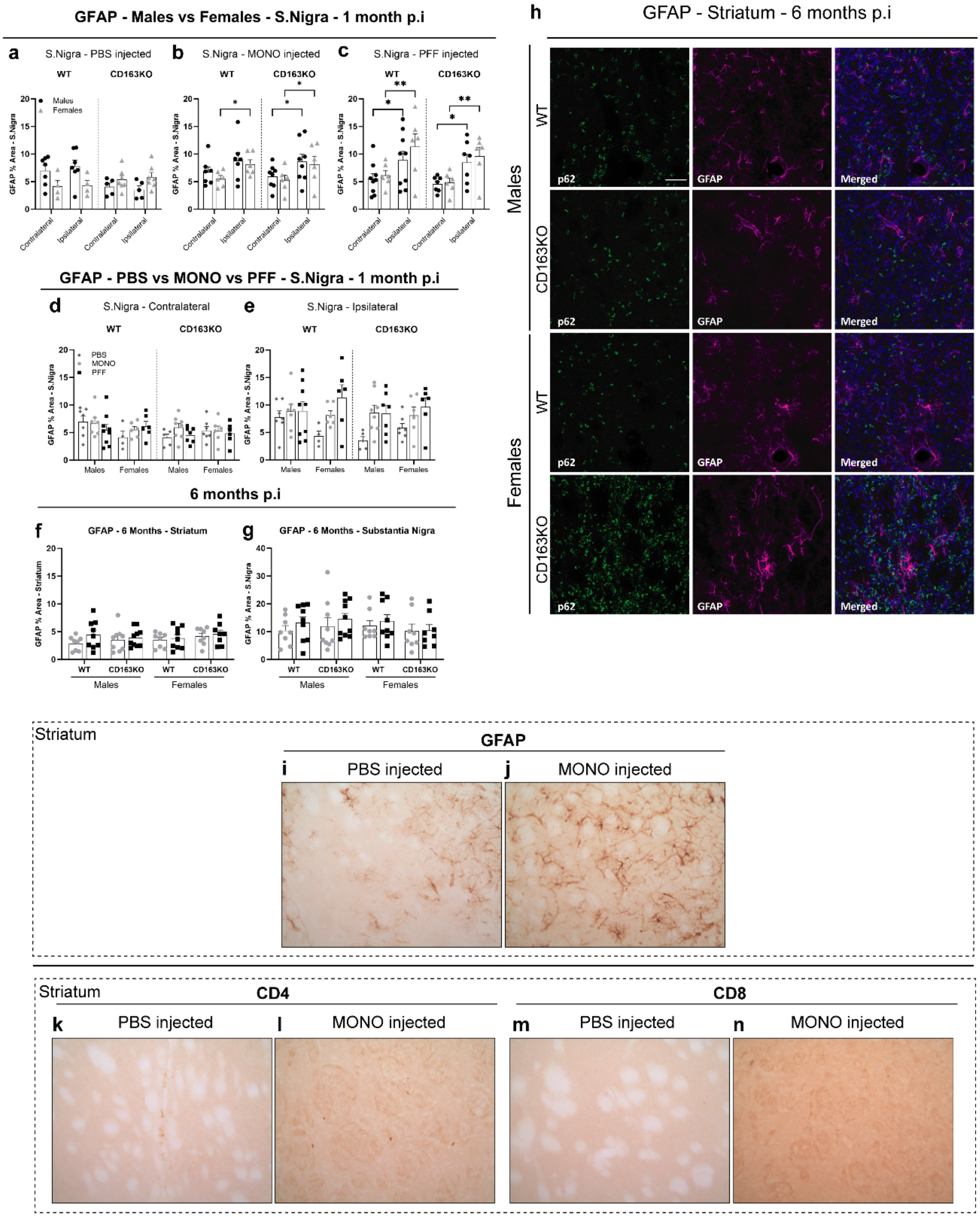

Supplementary Fig.7 GFAP expression after α-syn injection. Bar graphs with individual values represent the percentage of area covered by GFAP staining in the **d)** contralateral and **e)** ipsilateral SN 1 month post- **a)** PBS **b)** α-syn MONO and **c)** α-syn PFF injection; and in the **f)** ipsilateral striatum and **g)** ipsilateral SN 6 months post-α-syn MONO/PFF injection. **h)** Representative confocal images of p62 (green) labeling with GFAP (magenta) at 6 months p.i, displaying individual channels and merged/composite images (with DAPI (blue)) in the ipsilateral SN of α-syn PFF animals. **i-n)** Representative striatal images of GFAP, CD4 and CD8 immunostaining in **i,k,m)** PBS and **j,l,n)** α-syn MONO-injected animals at 1-month p.i.

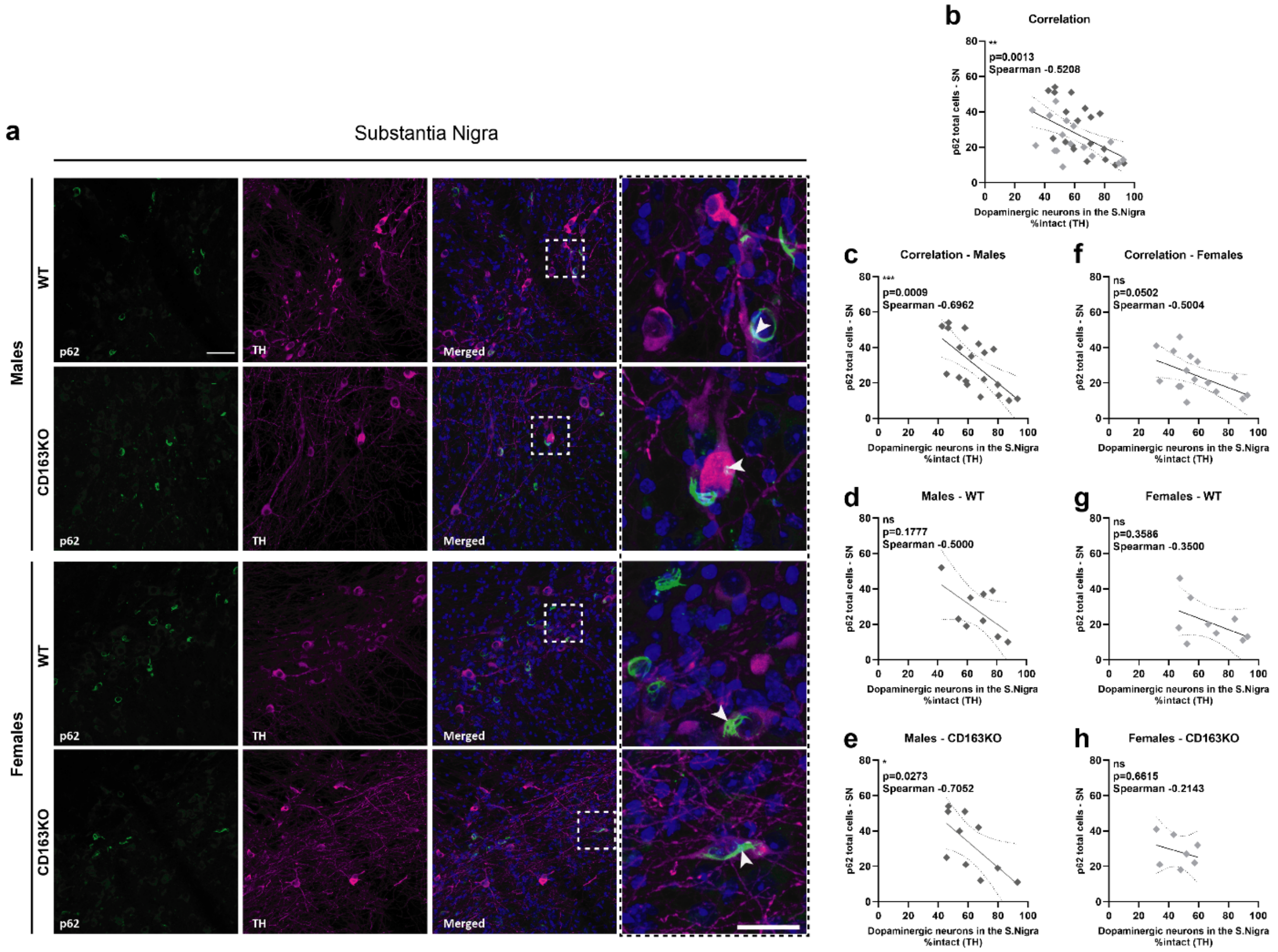

Supplementary Fig.8 p62 positive inclusions co-localize with TH in SN cell bodies and are associated with cell death in CD163KO males. a) Representative 20x view confocal images of TH (magenta) labeling with p62 (green) 6 months p.i, displaying individual channels and composite images (with DAPI (blue)) in the ipsilateral SN. Squares with white dashed lines delineate the cropped region of the amplified images. White arrowheads indicate co-localization. Scale bar represents 50 µm in the left panel images and 25 µm in the right cropped panel. **b-h)** Shows the correlation between the number of p62+ aggregates and the number of TH+ neurons in the SN of α-syn PFF animals. Statistics: Spearman two-tail p-values (*p<0.05, **<0.01, ***<0.001), Spearman r and best-fit slope with 95% confidence intervals are plotted.

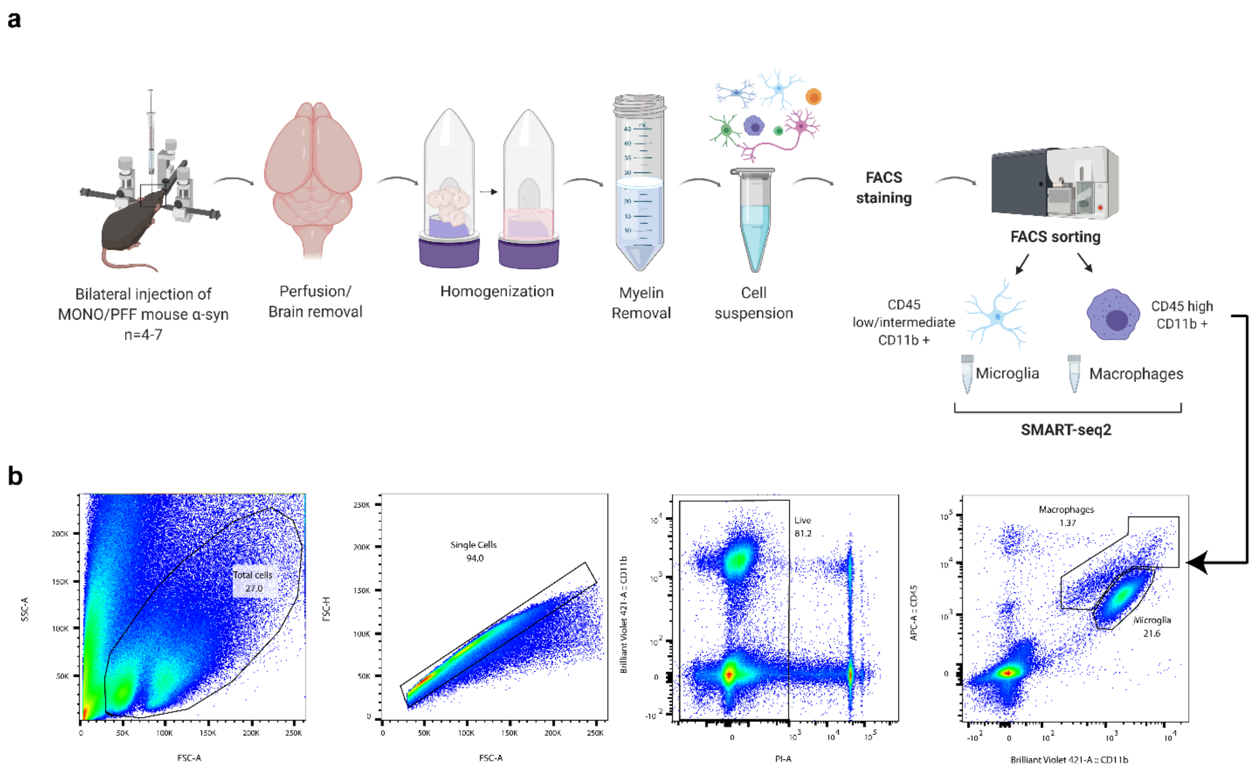

Supplementary Fig. 9 Flow cytometry gating strategy for Microglia and Macrophage populations. **a)** Image illustration of experimental study design of brain cells isolation for SMARTSeq2. **b)** Flow cytometry gating strategy for FACS sorting of Microglia (CD45 low/intermediate, CD11b positive) and Macrophage (CD45 high, CD11b positive) populations. Image created with BioRender.com

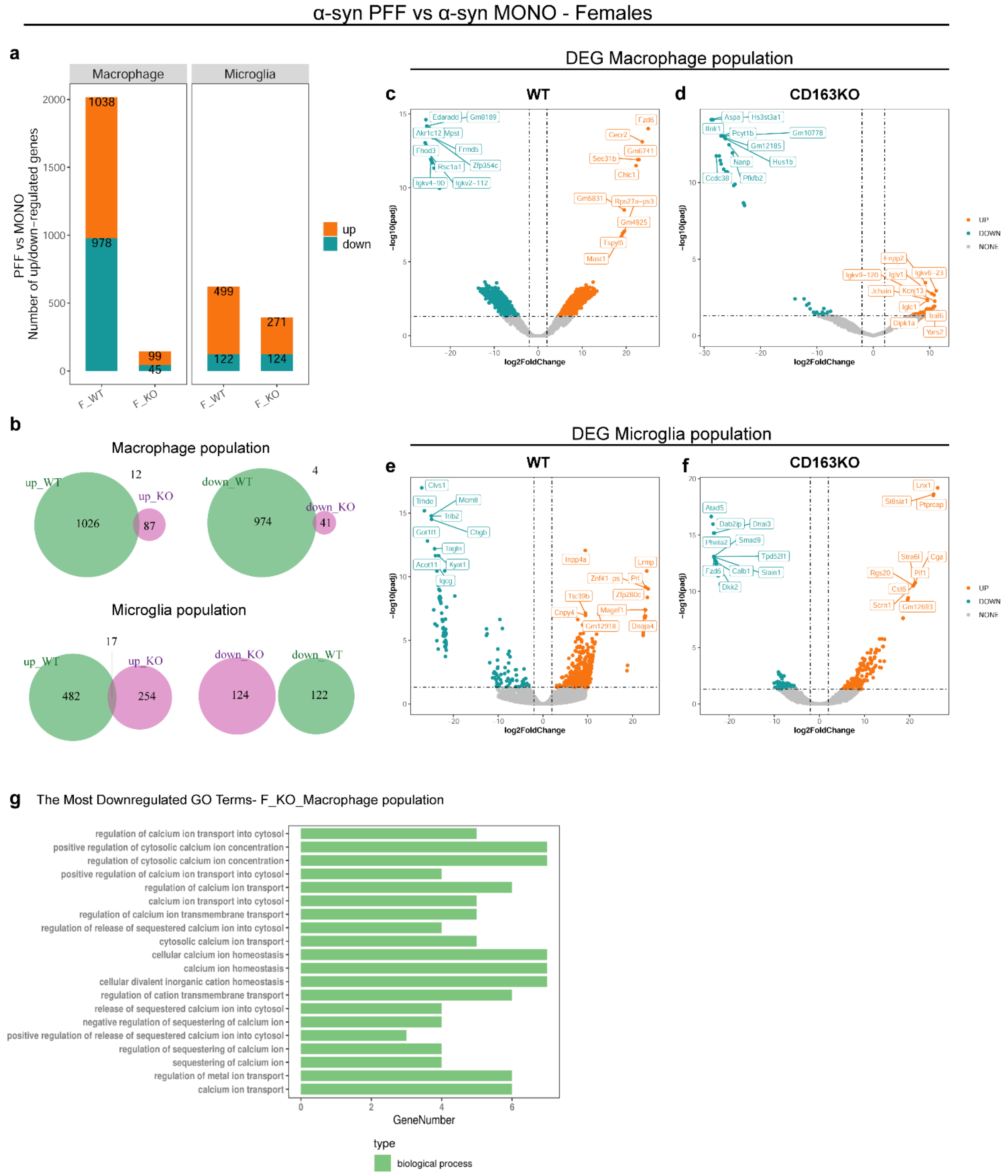

Supplementary Fig.10. Differentially expressed genes (DEGs) in Female α-syn PFF Macrophage and Microglia populations (vs. MONO). a) Bar graph showing the number of up/downregulated genes in α-syn PFF vs. MONO in Macrophage and Microglia populations 2 months p.i. **b)** Venn diagrams of shared DEGs. **c-f)** Volcano scatter-plots (-log10(padj) *vs* log2FoldChange) of DEGs in the Macrophage population and Microglia population. **g)** Gene Ontology (GO) analysis on the downregulated genes in CD163KO female macrophages.

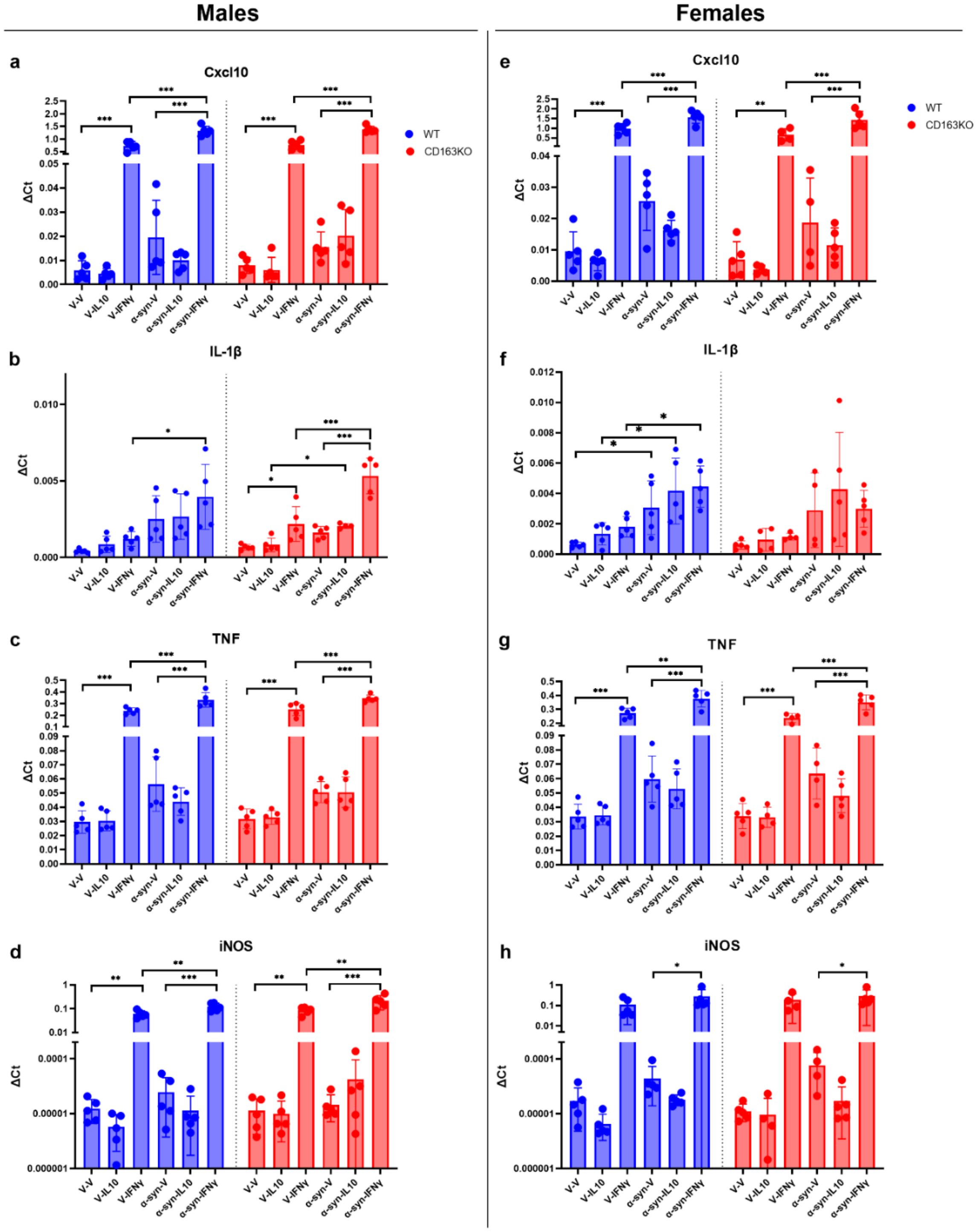

Supplementary Fig.11 Pro-inflammatory gene expression profile of BMDM from WT and CD163KO mice after *in vitro* stimulation and α-syn PFF treatment. Expression of M1 signature markers in *in vitro* stimulated BMDM isolated from WT and CD163KO **a-d)** males and **e-h)** females (n=4-5). BMDM were unstimulated (V=Vehicle) or stimulated with IL-10 and IFNγ 24h prior to α-syn PFF treatment (6h). Statistics: Values are Mean±SD. Two-way ANOVA or Mixed effects analysis with Bonferroni correction (<0.0071). *p<0.0071, **<0.00071, ***<0.000

References:

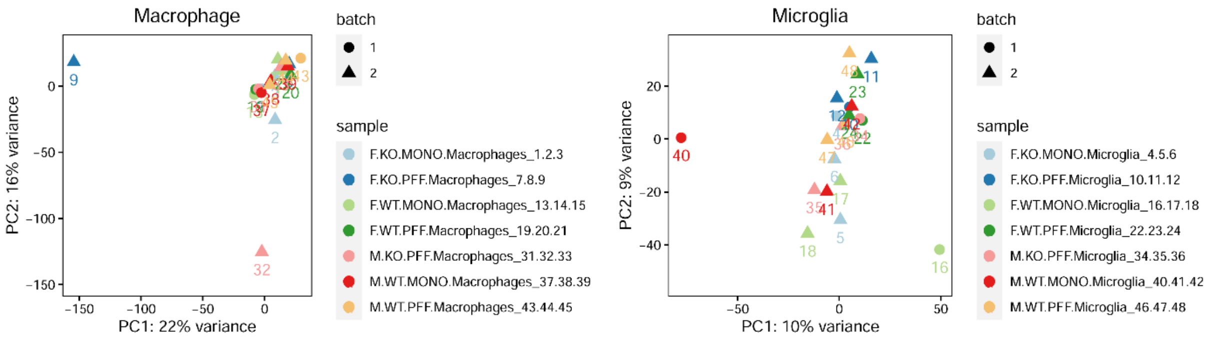

Supplementary Fig. 12 Principal component analysis (PCA) on Macrophage and Microglia populations. Batch effect was corrected and three outliers (Macrophage: #9 #32, Microglia: #16) were removed.
