# Supplementary material for: Sex-dimorphic neuroprotective effect of CD163 in an α-synuclein mouse model of Parkinson’s disease
Source: NPJ Parkinsons Dis. 2023 Dec 13;9:164. doi: 10.1038/s41531-023-00606-w (PMC10719342; doi:10.1038/s41531-023-00606-w)
Supplement: Supplementary file 2 — Related Manuscript File [file 41531_2023_606_MOESM2_ESM.pdf]

nature portfolio

Corresponding author(s): Marina Romero-ramos

Last updated by author(s): Nov 20, 2023

Reporting Summary

Nature Portfolio wishes to improve the reproducibility of the work that we publish. This form provides structure for consistency and transparency in reporting. For further information on Nature Portfolio policies, see our Editorial Policies and the Editorial Policy Checklist.

Statistics

For all statistical analyses, confirm that the following items are present in the figure legend, table legend, main text, or Methods section.

n/a	Confirmed	
☐	☒	The exact sample size (*n*) for each experimental group/condition, given as a discrete number and unit of measurement
☐	☒	A statement on whether measurements were taken from distinct samples or whether the same sample was measured repeatedly
☐	☒	The statistical test(s) used AND whether they are one- or two-sided<br>*Only common tests should be described solely by name; describe more complex techniques in the Methods section.*
☐	☒	A description of all covariates tested
☐	☒	A description of any assumptions or corrections, such as tests of normality and adjustment for multiple comparisons
☐	☒	A full description of the statistical parameters including central tendency (e.g. means) or other basic estimates (e.g. regression coefficient) AND variation (e.g. standard deviation) or associated estimates of uncertainty (e.g. confidence intervals)
☒	☐	For null hypothesis testing, the test statistic (e.g. *F*, *t*, *r*) with confidence intervals, effect sizes, degrees of freedom and *P* value noted<br>*Give P values as exact values whenever suitable.*
☒	☐	For Bayesian analysis, information on the choice of priors and Markov chain Monte Carlo settings
☒	☐	For hierarchical and complex designs, identification of the appropriate level for tests and full reporting of outcomes
☐	☒	Estimates of effect sizes (e.g. Cohen's *d*, Pearson's *r*), indicating how they were calculated

Our web collection on statistics for biologists contains articles on many of the points above.

Software and code

Policy information about availability of computer code

Data collection	NA
Data analysis	NA

For manuscripts utilizing custom algorithms or software that are central to the research but not yet described in published literature, software must be made available to editors and reviewers. We strongly encourage code deposition in a community repository (e.g. GitHub). See the Nature Portfolio guidelines for submitting code & software for further information.

Data

Policy information about availability of data

All manuscripts must include a data availability statement. This statement should provide the following information, where applicable:

- Accession codes, unique identifiers, or web links for publicly available datasets
- A description of any restrictions on data availability
- For clinical datasets or third party data, please ensure that the statement adheres to our policy

The authors confirm that the data supporting the findings of this study are available within the article and its supplementary material, or otherwise are available from the corresponding author, upon reasonable request. High-throughput RNA sequence data have been deposited to the Gene Expression Omnibus (Accession number GSE243536). Supplementary material is available online.

Research involving human participants, their data, or biological material

Policy information about studies with human participants or human data. See also policy information about sex, gender (identity/presentation), and sexual orientation and race, ethnicity and racism.

Reporting on sex and gender	NA
Reporting on race, ethnicity, or other socially relevant groupings	NA
Population characteristics	NA
Recruitment	NA
Ethics oversight	NA

Note that full information on the approval of the study protocol must also be provided in the manuscript.

Field-specific reporting

Please select the one below that is the best fit for your research. If you are not sure, read the appropriate sections before making your selection.

☒ Life sciences ☐ Behavioural & social sciences ☐ Ecological, evolutionary & environmental sciences

For a reference copy of the document with all sections, see nature.com/documents/nr-reporting-summary-flat.pdf

Life sciences study design

All studies must disclose on these points even when the disclosure is negative.

Sample size	Sample size was calculated based on previous published studies as stated in the manuscript
Data exclusions	Outliers were removed using statistical methods stated in the manuscript.
Replication	In vitro data was replicated. In vivo included a n number or animals
Randomization	Random
Blinding	Investigators were blinded to the groups identity while analyzing.

Reporting for specific materials, systems and methods

We require information from authors about some types of materials, experimental systems and methods used in many studies. Here, indicate whether each material, system or method listed is relevant to your study. If you are not sure if a list item applies to your research, read the appropriate section before selecting a response.

Materials & experimental systems

n/a	Involved in the study	
☐	☒	Antibodies
☐	☐	Eukaryotic cell lines
☐	☐	Palaeontology and archaeology
☐	☒	Animals and other organisms
☐	☐	Clinical data
☐	☐	Dual use research of concern
☐	☐	Plants

Methods

n/a	Involved in the study	
☐	☐	ChIP-seq
☐	☒	Flow cytometry
☐	☐	MRI-based neuroimaging

Antibodies

Antibodies used	Anti-tyrosine hydroxylase (TH polyclonal, 1:750, Millipore, AB152), anti-aggregated α-syn MJFR-14-6-4-2 (MJF14 Monoclonal,1:25000, Abcam, Ab209538), anti-phosphorylated α-syn (pSer129 α-syn Monoclonal/D1R1R, 1:3000, Cell Signaling Technology, #23706), MHCII (Monoclonal/M5/114.15.2, 1:400, eBioscience, 14-5321), p62/SQSTM1 (Polyclonal, 1:2000, Nordic Biosite, 18420-1-AP), CD68 (Monoclonal/FA-11, 1:1000, BioRad, MCA1957GA), Iba-1 (Polyclonal, 1:1000, Wako Fujifilm, 019-19741),

	GFAP (Polyclonal, 1:5000, Abcam, ab7260), CD4 (Monoclonal/RM4-5, 1:500, BD Biosciences, #553043), CD8 (Monoclonal/4SM15, 1:500, eBioscience, 14-0808-82), p62 protein C-terminus (Polyclonal, 1:1000 Nordic Biosite, 318-GP62-C)
Validation	Anti-TH Millipore (ab152) Validation:None provided; Anti-MJF14 (misfolded a-syn) Abcam (ab209538) Validation:None provided; Anti-pSer129 (phosphorylated a-syn) Cell signaling technologies (23706) Validation: Biological Strategies, Orthogonal; Anti- MHCII eBioscience (14-5321) Validation: Cell Treatment; Anti- P62/SQSTM1 Nordic Biosite (18420-1-AP) Validation:Knockdown; Anti- P62/SQSTM1 (c-terminus) Nordic Biosite (GP62-C) Validation:None provided; Anti- CD68 Biorad (MCA1957GA) Validation:None provided; Anti-GFAP Abcam (ab7260) Validation:None provided, Anti- Iba-1 Wako Fujifilm (019-19741) Validation:None provided; CD4(Monoclonal/RM4-5, 1:500, BD Biosciences, #553043) Validation: None provided; CD8 (Monoclonal/4SM15, 1:500, eBioscience, 14-0808-82) validation: immunohistochemistry of FFPA mouse tissue and flow cytometry of mouse splenocytes; p62 protein C-terminus (Polyclonal, 1:1000 Nordic Biosite, 318-GP62-C) validation: None provided

Eukaryotic cell lines

Policy information about cell lines and Sex and Gender in Research

Cell line source(s)	*State the source of each cell line used and the sex of all primary cell lines and cells derived from human participants or vertebrate models.*
Authentication	*Describe the authentication procedures for each cell line used OR declare that none of the cell lines used were authenticated.*
Mycoplasma contamination	*Confirm that all cell lines tested negative for mycoplasma contamination OR describe the results of the testing for mycoplasma contamination OR declare that the cell lines were not tested for mycoplasma contamination.*
Commonly misidentified lines (See ICLAC register)	*Name any commonly misidentified cell lines used in the study and provide a rationale for their use.*

Palaeontology and Archaeology

Specimen provenance	*Provide provenance information for specimens and describe permits that were obtained for the work (including the name of the issuing authority, the date of issue, and any identifying information). Permits should encompass collection and, where applicable, export.*
Specimen deposition	*Indicate where the specimens have been deposited to permit free access by other researchers.*
Dating methods	*If new dates are provided, describe how they were obtained (e.g. collection, storage, sample pretreatment and measurement), where they were obtained (i.e. lab name), the calibration program and the protocol for quality assurance OR state that no new dates are provided.*

☐ Tick this box to confirm that the raw and calibrated dates are available in the paper or in Supplementary Information.

Ethics oversight	*Identify the organization(s) that approved or provided guidance on the study protocol, OR state that no ethical approval or guidance was required and explain why not.*

Note that full information on the approval of the study protocol must also be provided in the manuscript.

Animals and other research organisms

Policy information about studies involving animals; ARRIVE guidelines recommended for reporting animal research, and Sex and Gender in Research

Laboratory animals	Adult male and female (12 weeks old) CD163KO (CD163tm1.1(KOMP)Vlcg) mice and WT (C57BL/6N) littermates (3-4 months old) (n=179 total (n=95 males (50 WT + 45 CD163KO); n=84 females (43 WT+ 41 CD163KO))
Wild animals	NA
Reporting on sex	Sex differences are a focus in the study, thus sex was used as a variable.
Field-collected samples	NA
Ethics oversight	All animal experiments were approved and performed under humane conditions in accordance with the ethical guidelines established by the Danish Animal Inspectorate and following EU legislation.

Note that full information on the approval of the study protocol must also be provided in the manuscript.

Clinical data

Policy information about clinical studies

All manuscripts should comply with the ICMJE guidelines for publication of clinical research and a completed CONSORT checklist must be included with all submissions.

Clinical trial registration	*Provide the trial registration number from ClinicalTrials.gov or an equivalent agency.*
Study protocol	*Note where the full trial protocol can be accessed OR if not available, explain why.*
Data collection	*Describe the settings and locales of data collection, noting the time periods of recruitment and data collection.*
Outcomes	*Describe how you pre-defined primary and secondary outcome measures and how you assessed these measures.*

Dual use research of concern

Policy information about dual use research of concern

Hazards

Could the accidental, deliberate or reckless misuse of agents or technologies generated in the work, or the application of information presented in the manuscript, pose a threat to:

No	Yes	
☐	☐	Public health
☐	☐	National security
☐	☐	Crops and/or livestock
☐	☐	Ecosystems
☐	☐	Any other significant area

Experiments of concern

Does the work involve any of these experiments of concern:

No	Yes	
☐	☐	Demonstrate how to render a vaccine ineffective
☐	☐	Confer resistance to therapeutically useful antibiotics or antiviral agents
☐	☐	Enhance the virulence of a pathogen or render a nonpathogen virulent
☐	☐	Increase transmissibility of a pathogen
☐	☐	Alter the host range of a pathogen
☐	☐	Enable evasion of diagnostic/detection modalities
☐	☐	Enable the weaponization of a biological agent or toxin
☐	☐	Any other potentially harmful combination of experiments and agents

Plants

Seed stocks	*Report on the source of all seed stocks or other plant material used. If applicable, state the seed stock centre and catalogue number. If plant specimens were collected from the field, describe the collection location, date and sampling procedures.*
Novel plant genotypes	*Describe the methods by which all novel plant genotypes were produced. This includes those generated by transgenic approaches, gene editing, chemical/radiation-based mutagenesis and hybridization. For transgenic lines, describe the transformation method, the number of independent lines analyzed and the generation upon which experiments were performed. For gene-edited lines, describe the editor used, the endogenous sequence targeted for editing, the targeting guide RNA sequence (if applicable) and how the editor was applied.*
Authentication	*Describe any authentication procedures for each seed stock used or novel genotype generated. Describe any experiments used to assess the effect of a mutation and, where applicable, how potential secondary effects (e.g. second site T-DNA insertions, mosiacism, off-target gene editing) were examined.*

ChIP-seq

Data deposition

☐ Confirm that both raw and final processed data have been deposited in a public database such as GEO.

☐ Confirm that you have deposited or provided access to graph files (e.g. BED files) for the called peaks.

Data access links<br>*May remain private before publication.*	*For "Initial submission" or "Revised version" documents, provide reviewer access links. For your "Final submission" document, provide a link to the deposited data.*
Files in database submission	*Provide a list of all files available in the database submission.*
Genome browser session<br>(e.g. UCSC)	*Provide a link to an anonymized genome browser session for "Initial submission" and "Revised version" documents only, to enable peer review. Write "no longer applicable" for "Final submission" documents.*

Methodology

Replicates	*Describe the experimental replicates, specifying number, type and replicate agreement.*
Sequencing depth	*Describe the sequencing depth for each experiment, providing the total number of reads, uniquely mapped reads, length of reads and whether they were paired- or single-end.*
Antibodies	*Describe the antibodies used for the ChIP-seq experiments; as applicable, provide supplier name, catalog number, clone name, and lot number.*
Peak calling parameters	*Specify the command line program and parameters used for read mapping and peak calling, including the ChIP, control and index files used.*
Data quality	*Describe the methods used to ensure data quality in full detail, including how many peaks are at FDR 5% and above 5-fold enrichment.*
Software	*Describe the software used to collect and analyze the ChIP-seq data. For custom code that has been deposited into a community repository, provide accession details.*

Flow Cytometry

Plots

Confirm that:

☒ The axis labels state the marker and fluorochrome used (e.g. CD4-FITC).

☒ The axis scales are clearly visible. Include numbers along axes only for bottom left plot of group (a 'group' is an analysis of identical markers).

☒ All plots are contour plots with outliers or pseudocolor plots.

☒ A numerical value for number of cells or percentage (with statistics) is provided.

Methodology

Sample preparation	Animals were transcardially perfused through the ascending aorta with ice-cold PBS, brains were extracted and cells isolated using the Adult Brain Dissociation Kit (Miltenyi Biotec) in accordance with the manufacturer's protocol. Briefly, brains were cut into approximately 0.5 cm pieces and transferred into gentleMACS C Tubes containing the provided enzymes. Tissue was dissociated using the gentleMACS Octo Dissociator with Heaters for 30 min in the appropriate program. Homogenates were filtered through 70µm cell strainers and the cell suspension was centrifuged at 300xg, 10 minutes at 4°C. Cells were resuspended in a Debris Removal Solution (in DPBS containing CaCl2, MgCl2, 1g/L D-Glucose, and 36mg/L Pyruvate, Gibco) and centrifuged at 4°C and 3000xg for 10 minutes. Gradient centrifugation formed three phases and the top two layers containing myelin debris were removed. Cells were washed with 1x PBS and counted in the MOXI Z Mini Automated cell counter (ORFLO).<br>Freshly isolated brain cells were blocked with CD16/CD32 (clone 2.4G2, Mouse BD Fc block, BD Pharming) and 10% goat serum in PBS for 10min at 4°C. Single-cell suspensions were incubated in darkness at 4°C for 30 min with CD11b antibody conjugated to BV421 dye (50µg/mL, clone M1/70, BioLegend) and CD45 antibody conjugated to APC dye (0.2mg/mL, clone 30-F11, BioLegend). Cells were washed with PBS and 0.5% BSA, centrifuged at 4°C and 400xg for 5 minutes, and resuspended in cold PBS. Propidium iodide (PI) was added to the cell suspension for identification of dead cells.
Instrument	FACSAria III high speed cell sorter (BD Biosciences, San Jose, CA)
Software	FlowJo v10
Cell population abundance	Microglia (200000-500000 cells) and macrophage (10000-30000 cells) cell suspensions (>90% purity after sorting)
Gating strategy	Identification of total cells and exclusion of debris was performed based on SSC-A vs. FSC-A, followed by doublet exclusion by

Gating strategy

FSC-A vs. FSC-H. Exclusion of dead cells was done based on PI-A vs. CD11b-BV421-A. Microglia (CD11b+/CD45low/intermediate) and macrophages (brain border-associated and infiltrated, neutrophils and NK cells, CD11b+/CD45high) were identified based on CD45-APC-A vs. CD11b-BV421-A

☒ Tick this box to confirm that a figure exemplifying the gating strategy is provided in the Supplementary Information.

Magnetic resonance imaging

Experimental design

Design type

Indicate task or resting state; event-related or block design.

Design specifications

Specify the number of blocks, trials or experimental units per session and/or subject, and specify the length of each trial or block (if trials are blocked) and interval between trials.

Behavioral performance measures

State number and/or type of variables recorded (e.g. correct button press, response time) and what statistics were used to establish that the subjects were performing the task as expected (e.g. mean, range, and/or standard deviation across subjects).

Acquisition

Imaging type(s)

Specify: functional, structural, diffusion, perfusion.

Field strength

Specify in Tesla

Sequence & imaging parameters

Specify the pulse sequence type (gradient echo, spin echo, etc.), imaging type (EPI, spiral, etc.), field of view, matrix size, slice thickness, orientation and TE/TR/flip angle.

Area of acquisition

State whether a whole brain scan was used OR define the area of acquisition, describing how the region was determined.

Diffusion MRI ☐ Used ☐ Not used

Preprocessing

Preprocessing software

Provide detail on software version and revision number and on specific parameters (model/functions, brain extraction, segmentation, smoothing kernel size, etc.).

Normalization

If data were normalized/standardized, describe the approach(es): specify linear or non-linear and define image types used for transformation OR indicate that data were not normalized and explain rationale for lack of normalization.

Normalization template

Describe the template used for normalization/transformation, specifying subject space or group standardized space (e.g. original Talairach, MNI305, ICBM152) OR indicate that the data were not normalized.

Noise and artifact removal

Describe your procedure(s) for artifact and structured noise removal, specifying motion parameters, tissue signals and physiological signals (heart rate, respiration).

Volume censoring

Define your software and/or method and criteria for volume censoring, and state the extent of such censoring.

Statistical modeling & inference

Model type and settings

Specify type (mass univariate, multivariate, RSA, predictive, etc.) and describe essential details of the model at the first and second levels (e.g. fixed, random or mixed effects; drift or auto-correlation).

Effect(s) tested

Define precise effect in terms of the task or stimulus conditions instead of psychological concepts and indicate whether ANOVA or factorial designs were used.

Specify type of analysis: ☐ Whole brain ☐ ROI-based ☐ Both

Statistic type for inference

(See Eklund et al. 2016)

Specify voxel-wise or cluster-wise and report all relevant parameters for cluster-wise methods.

Correction

Describe the type of correction and how it is obtained for multiple comparisons (e.g. FWE, FDR, permutation or Monte Carlo).

Models & analysis

n/a	Involved in the study
☐	☐ Functional and/or effective connectivity
☐	☐ Graph analysis
☐	☐ Multivariate modeling or predictive analysis

Functional and/or effective connectivity	*Report the measures of dependence used and the model details (e.g. Pearson correlation, partial correlation, mutual information).*
Graph analysis	*Report the dependent variable and connectivity measure, specifying weighted graph or binarized graph, subject- or group-level, and the global and/or node summaries used (e.g. clustering coefficient, efficiency, etc.).*
Multivariate modeling and predictive analysis	*Specify independent variables, features extraction and dimension reduction, model, training and evaluation metrics.*
